# A comparison of contact patterns derived from the population structure in agent-based models and empirical contact survey data

**DOI:** 10.1371/journal.pcbi.1013533

**Published:** 2026-06-18

**Authors:** Janik Suer, Johannes Ponge, Michael Brüggemann, Jan Pablo Burgard, Vitaly Belik, Bernd Hellingrath, Alejandra Rincón Hidalgo, Andrzej K. Jarynowski, Richard Pastor, Huynh Thi Phuong, Steven Schulz, Ashish Thampi, Chao Xu, Marlli Zambrano, Rafael Mikolajczyk, André Karch, Veronika K. Jaeger, on behalf of the OptimAgent Consortium

**Affiliations:** 1 Institute of Epidemiology and Social Medicine, University of Münster, Münster, Germany; 2 Interdisciplinary Center for Mathematical Modeling of Infectious Disease Dynamics (IMMIDD), University of Münster, Münster, Germany; 3 Department of Information Systems, University of Münster, Münster, Germany; 4 Department of Economic and Social Statistics, Trier University, Trier, Germany; 5 System Modelling Group, Institute of Veterinary Epidemiology and Biostatistics, Freie Universität Berlin, Berlin, Germany; 6 Machine Learning Unit, Department of Engineering, NET CHECK GmbH, Berlin, Germany; 7 Institute for Medical Epidemiology, Biometrics, and Informatics (IMEBI), Interdisciplinary Center for Health Sciences, Medical Faculty of the Martin Luther University Halle-Wittenberg, Halle, Germany; 8 Middle German Center for Biomedical Modelling and Simulation in Life Sciences (CeMMSiS), Halle, Germany; Queen Mary University of London, UNITED KINGDOM OF GREAT BRITAIN AND NORTHERN IRELAND

## Abstract

Agent-based models (ABMs) are powerful tools for simulating disease spread, relying on individual-level interaction rules from which emergent dynamics arise. An important component in ABMs is contact behaviour. To reduce computational complexity, contact behaviour in ABMs is often assumed as random mixing within structurally defined settings (as, e.g., workplaces). with setting composition typically based on empirical data such as census information. However, the validity of this approach to represent contacts remains unclear. To address this gap, we compare the contact structure derived through this approach in a large-scale ABM with empirical contact survey data with respect to age contact matrices for households, schools, workplaces, all remaining contact settings, and all contacts combined (based on difference matrices and sum of squared errors (SSE)). Our results demonstrate that random mixing in settings with known age compositions like households (SSE:0.7(95%CI0.4–0.9)), schools (SSE:0.7(95%CI:0.3–1.1)) and workplaces (SSE:0.5(95%CI:0.2-0.7)), captures basic interaction patterns but fails to account for age-related variation in contact numbers. The largest differences arise for contacts outside these settings (SSE:3.8(95%CI:1.2–6.5)), as ABMs typically use random regional contacts that do not capture age-structured behaviour observed in contact surveys. Applying contact matrices from both approaches to an age-structured compartmental model, leads to noticeable differences in simulated epidemic outcomes regarding reproduction numbers and spreading dynamics between age groups. Our results suggest that naïve approaches to represent contact behaviour in ABMs based on population structure can be valid in settings with defined age-structures while settings with low a priori structure require more advanced methods to represent contact behaviour observed in contact surveys.

## Author summary

Agent-based infectious disease models are tools for understanding disease spread and evaluating potential countermeasures. These models simulate the behaviour of individuals based on simple rules and allow a detailed representation of disease transmissions. One essential rule is the contact behaviour of individuals, as it determines which pathogen transmissions are possible. Agent-based models often assume randomly mixing individuals in their locations, such as households or workplaces, which are simulated based on the overall population structure. We investigate if this approach gives a realistic representation of contact patterns by comparing the contact structure simulated in an agent-based model reflecting the German population with contact data gathered in a German contact survey. We find that random mixing can serve as an approximation for settings such as household, schools, and workplaces where the contact structure is similar to the age structure of the individuals present. For contacts outside pre-defined population structures, contact patterns derived from population information depart from empirical survey data. Using a simple compartmental model, we concluded that the observed differences could lead to epidemiologically relevant differences in simulated pathogen spread. However, further studies are required to estimate the effect of different contact structures within the agent-based models.

## Introduction

Understanding the complex dynamics of disease spread requires tools capable of capturing individual-level behaviours and interactions. Agent-based models (ABMs) provide this capability, enabling the simulation of epidemics in realistic populations. The accuracy of ABMs for modelling disease spread relies on the accurate specification of individual behaviours, in particular the contact behaviour. Depending on the type of pathogen considered, the contacts that models must be able to represent can vary. For instance, when modelling respiratory infections such as COVID-19, face-to-face contacts between individuals arising from, e.g., conversing with or sitting near other people are the most important types of contact that models must be able to represent.

The real-world contact behaviour of individuals has been studied frequently on different scales in various populations [1–4]. The most well-known study on individuals’ contact behaviour is the POLYMOD survey [2], which assessed the contact behaviour of individuals in multiple European countries and has been used to parametrise infectious disease models over many years [5–8]. However, as the contact behaviour of individuals has potentially changed over the years, newer studies should be considered [9]. The COVIMOD study is one such recent study that assessed the contact behaviour of individuals during and shortly after the COVID-19 pandemic in Germany [4,10–12].

While ABMs can theoretically model contact behaviour with arbitrary complexity, computational limitations require the use of simple approaches, to enable efficient simulation. These considerations are particularly relevant for models that aim to simulate large populations [6,13–16]. One modelling framework for such models is GEMS (German Epidemic Microsimulation System), which facilitates the simulation of disease dynamics in the entire population of Germany [14,15]. While GEMS supports various contact sampling methods, the most efficient method for simulating the entire German population assumes a randomly mixing population within settings. This assumption of random mixing in settings is used in various large-scale ABMs to decrease the computational complexity for large populations and will be the focus of our analysis [5,6,16,17].

In the base parametrisation of GEMS, pre-defined settings correspond to the individuals’ household, workplace, school, and municipality. Given the population structure, the simulated contact structure does not correspond to random mixing of the entire population but to a random mixing of the individuals associated with the same setting, imposing the age-structure of the setting as its contact structure. The age-structure of the settings is created during the development of the synthetic population. Here, demographic and setting-specific data such as census and workplace data are being used to create individuals and associate them with the appropriate settings. Using random mixing in the settings yields a computationally efficient model but may not be capable of representing the nuanced contact structure observed in the real-world. This raises the central question: *Can the assumption of random mixing within settings reproduce the contact behaviour observed in empirical contact surveys?*

While this question is particularly relevant to large-scale ABMs for infectious disease modelling, which usually rely on the assumption of random mixing to reduce computational complexity, it is also relevant in other fields [5,6]. Random mixing assumptions are commonly employed when constructing contact networks and matrices from synthetic population data [18–22]. Since such networks and matrices are used in infectious disease models, including compartmental and network models, assessing the validity of the random mixing assumption has implications for infectious disease modelling practice beyond large-scale ABMs.

Thus, to assess the random mixing assumption, we compare the contact structure simulated in GEMS using the random mixing contact sampling method with empirical contact data from the COVIMOD survey. The comparison involves three steps. First, we extract the contact matrices from the ABM and the contact survey. Second, we calibrate the contact sampling method in the ABM by minimizing the difference between the simulated and the survey-based contact matrix for each setting. Third, we investigate the remaining structural differences and identify their origins. We perform our analysis for the household, workplace, school, and the other contacts (all contacts outside of the households, workplaces, and schools) and all contacts combined. Lastly, we assess which epidemic impact the differences in contact matrices would have in an age-stratified compartmental Susceptible-Infectious-Resistant (SIR) model.

## Methods

### Data

The COVIMOD study is a longitudinal cohort study designed to capture the contact behaviour of the German population during and shortly after the COVID-19 pandemic. The study consists of 36 waves during which a demographically representative sample of the German population completed a questionnaire regarding themselves and their contacts on the preceding day. To capture child contacts, a subset of adults with children under the age of eighteen completed the questionnaire as proxies for their children. Further details can be found elsewhere [4,10–12]. Since we want to consider the contact behaviour under non-restrictive conditions, we use the final three survey waves (wave 34 – 36), conducted between November 2022 and March 2023 and consider them as one large survey, treating participants who responded to multiple survey waves as independent participants. Since all survey waves were conducted during the same winter period and no contact restrictions were imposed, we neglected seasonal effects and other substantial changes in contact behaviour. These three waves capture the contacts of 7,481 participants, who reported a total of 23,146 individual contacts categorized as household (8,056), workplace (4,495), school (2,370) and other contacts (8,224), i.e., contacts the participant had in their household, workplace, school or in any other place, respectively. Note that while COVIMOD includes more contact settings, we group them together as “other contacts” to allow for an easier comparison with the contact patterns derived from the ABM via the population structure.

Prior to the analysis, we imputed missing values for participants’ age. For participants that only supplied their age group the exact age was imputed using a weighted sampling based on the 2023 age-distribution in Germany [23].

In the COVIMOD survey, participants could also report group contacts in settings other than the household. For these group contacts, participants had to report the number of individuals they had met and their collective age group. These age groups were defined as under 18, 18–64, and 65 or over. However, due to the coarse resolution of these age groups, obtaining accurate mixing matrices is not trivial. Since participants only reported these coarse collective age groups, it is not possible to construct the contact matrices with five-year age bands from group contacts and individual contacts combined directly. While it would be possible to map the individual contacts reported in five-year age bands to the age groups of the group contacts, doing the opposite requires the imputation of the age groups of the group contact partners. There are different approaches to impute the age groups of the group contacts; however, they need to be based on assumptions to generate the more granular age groups [24,25]. As the COVIMOD study without group contacts already includes a large sample of contacts and we are more interested in the structure of the represented contacts than the actual number of contacts we consider only individually reported contacts and exclude group contacts in the main analyses. However, we present analogous analyses using two different methods for imputing the age of group contacts in S1 Appendix. This allows us to assess the impact of our decision to exclude group contacts from the analysis.

Contact matrices for the four contact categories (household, workplace, school and other) were generated from the COVIMOD data using the *socialmixr* package [26], a package for the R programming language [27] commonly used for creating contact matrices based on contact survey data for compartmental models. The entries $m_{ij}$ of these contact matrices represent the average number of contacts reported by individuals in age group $A_{i}$ with individuals in age group $A_{j}$ during a 24-hour period in the given setting. Here, i and j are indices iterating over the age groups $i,j\in \{\text{1},\ldots ,N_{age}\}$. We defined age groups using five-year right-open intervals with the last age group being 80 + such that $N_{age}=\text{1}\text{7}$. To account for variations in contact behaviour between weekends and weekdays, we weighted contacts based on whether they were reported on a weekday (weight prop. to 5/7) or on a weekend (weight prop. to 2/7) to obtain the contact behaviour for an “average” day.

Contacts between people are in general symmetric, meaning if person A has a contact with person B, B also has a contact with A. This symmetry can be expressed as:


$$\begin{array}{c} m_{ij}N_{i}=m_{ji}N_{j}, \end{array}$$

where $m_{ij}$ and $m_{ji}$ represent the corresponding entries of the contact matrix and $N_{i}$ and $N_{j}$ the size of the age groups i and j, respectively. We used this symmetry in the socialmixr package when constructing the contact matrices for household contacts, other contacts, and all combined contacts. However, this symmetry does not hold for schools and workplaces due to the possibility of participants reporting the same contact in different settings. For example, while a teacher will report a teacher-student contact as a workplace contact, a student will report the same contact as a school contact. Consequently, the symmetry condition was not employed for the contact matrices of workplaces and schools. In structured ABMs, household contacts per definition typically represent only contacts with household members. Contacts at home with non-household members in COVIMOD were therefore reclassified as contacts in municipalities.

Since in COVIMOD a fraction of participants with children were asked to fill out the form as proxies for their children and not for themselves therefore disturbing the originally representative sampling, the symmetry was employed to mitigate the underrepresentation of adults living with children in the survey,

### GEMS

GEMS (German Epidemic Microsimulation System) is an ABM that allows the simulation of disease spread within an entire population (in this case using the population structure of Germany). In GEMS individuals are associated with settings in which they can have contacts [14]. Therefore, the potential contact partners of a person are the other members of the settings the person is associated with. The settings taken into account in this analysis are households, workplaces, schools, and municipalities, with the latter simulating all contacts outside of households, workplaces, and schools. Schools and workplaces consist of four hierarchical substructures. For the schools, these substructures are the school complex, schools, school years and classes, where each includes at least one of the latter. Similarly, the workplaces consist of workplace sites, workplaces, departments, and offices. Note that when we refer to school or workplace contacts, we refer to all contacts arising in any of the corresponding substructures. Otherwise, we will specifically refer to the substructures. In each of these settings, including the substructures of schools and workplaces, individuals’ contacts are sampled based on a user-defined contact sampling method.

While GEMS includes various contact sampling methods, we assess here the most efficient method, which assumes a randomly mixing population within settings. This method is common in large-scale ABMs since it ensures a feasible simulation of large populations [5,6,16,17]. In the following we denote this contact sampling method as random contact sampling.

The contact sampling for a specific individual I in the setting $S_{T}$ of type T works in two steps. First, the number of contacts $N_{C}$ the individual I will have is drawn from a Poisson distribution with mean $\lambda_{T}$, depending on the type T of the setting $S_{T}$. This means and distributions are based on the empirical data from COVIMOD so that the marginal number of contacts are stable across approaches. Second, from the individuals present in the setting $S_{T}$ excluding individual I, $N_{C}$ individuals are randomly drawn with replacement and recorded as contacts. Sampling with replacement is performed to reduce computational complexity. For large settings such as municipalities, where only a small proportion of individuals present are selected as contacts, sampling without replacement would result in the computational cost scaling with the number of individuals present rather than the number of contacts sampled [28]. For our analysis, contacts are drawn for randomly selected individuals in the population until the total number of $10^{7}$ recorded contacts are reached. This number of contacts was chosen as a cut-off to assure that uncertainties of the simulated contact behaviour are negligible for further analyses. For every selected individual all contacts they have during one day are sampled. Based on sampled contacts, we derive the simulated contact matrices. Since we know the exact ages of all contact partners, we can directly construct the contact matrices by grouping them into five-year age groups and calculating the average number of daily contacts between all age groups for every setting.

The structure of contacts in GEMS is a result of the association of individuals to settings. The associations with settings are determined during the generation of the Gesyland population [29,30], a synthetic population that represents various characteristics of the German population. The locations and types of settings, such as home locations, schools or workplaces, are obtained from OpenStreetMap [31]. The home locations are filled with households, where the household size is obtained from the household size distribution based on the German Census 2011 [32]. This Census data is available at a 100m square grid, allowing for regional variations in household size distribution. Individuals in the households are created probabilistically based on demographic tables within household size classes. Further variables like education, occupation and health related variables obtained from the German microcensus 2019 [33] and the German health survey GEDA (2019/2020-EHIS) [34], and added via a predictive random forest procedure. The assignment of individuals to schools and workplaces is based on individual characteristics and the interregional commuting structure derived from commuting tables [35]. Substructures in schools are created based on the structure of the school system. Schools consist of school years which consist of classes. Additionally multiple school may share the same common facilities and are structured in a school complex. For workplaces substructures are determined by randomly creating smaller groups of the workers in a workplace and thereby create departments and offices. Workplaces sharing the same location are grouped in a workplace site. While producing the individual properties and affiliations, the known constraints on higher levels are kept. These constraints included among others the total number of employed people in a region and the number of people having a certain educational level. Hence, the hierarchical population structure is consistent to known totals obtained from the German Census 2011 [32] regarding the households’ structure, and consistent to estimated totals for all other variables according to the German microcensus 2019 [33] and German health survey GEDA (2019/2020-EHIS) [34]. A dedicated description of the Gesyland population and the method used for its creation elsewhere [30].

While we use GEMS for our comparison, other ABMs follow the same methodology: associating population members to specific locations, in which random contacts are modelled [6,16,17,36]. As in GEMS, the included settings commonly correspond to households, workplaces, schools, and a regional setting that facilitates all other contacts. Models that employ this structure benefit from a simplified computation of contacts during the simulation by shifting the work from the contact sampling during the simulation to the creation of an accurate synthetic population before the simulation.

### Calibration and comparison

As the initial parametrisation of the random contact sampling in the ABM, the average number of contacts per day is set to one, i.e., $\lambda_{T}=\text{1}$ for all setting types T in the simulation. Based on the simulated contact matrix $\overset{T}{\underset{S}{M}}$ and the contact matrix derived from COVIMOD $\overset{T}{\underset{C}{M}}$ the average number of contacts $\lambda_{T}$ is calibrated as follows. Note that to simplify the notation, from now on we drop the setting type index T, while still referring to setting type specific variables. Since the entries in the contact matrix scale linearly with the defined average number of contacts λ, scaling the average number of contacts by α, such that $\lambda^{\prime }=\alpha \lambda$ results in a linearly scaled contact matrix $\overset{\prime }{\underset{S}{M}}=\alpha M_{S}$. As we want to align the contact structure in the ABM with that observed in the contact survey, we choose to minimize the difference between the two contact matrices to obtain the calibrated contact numbers $\lambda^{\prime }$ for the ABM. To describe the difference between the two contact matrices, we use the sum of squared errors (SSE), placing more emphasis on large deviations than small ones. The SSE for the survey-based contact matrix $M_{C}$ and the linearly scaled simulated contact matrix $M_{S}^{\prime }=\alpha M_{S}$ can be expressed as:


$$\begin{array}{c} SSE(M_{C},\;\overset{\prime }{\underset{S}{M}})=SSE(M_{C},\;\alpha M_{S})={\sum_{i,j}\left(\overset{C}{\underset{ij}{m}}-\alpha \overset{S}{\underset{ij}{m}}\;\right)}^{\text{2}}\; \end{array}$$ (1)

Although the unit of the SSE is number of contacts squared, we will treat it as a dimensionless number for brevity. Equation 1 is minimized at:


$$\begin{array}{c} \alpha =\;\frac{\sum_{i,j}\left(\overset{C}{\underset{ij}{m}}\overset{S}{\underset{ij}{m}}\;\right)}{\sum_{i,j}{\left(\overset{S}{\underset{ij}{m}}\;\right)}^{\text{2}}} \end{array}$$

Calculating α yields the optimal average number of contacts in the ABM for settings such as households, without further substructure. However, for settings which have substructures, i.e., schools and workplaces, the simulated contact matrix is equal to the sum of the contact matrices of the substructures which can be scaled individually, i.e., each substructure has its own λ-value. Therefore, the SSE can be expressed as:


$$\begin{array}{c} SSE\left(M_{C},\sum_{k}\alpha_{k}\overset{k}{\underset{S}{M}}\;\right)={\sum_{i,j}\left(\overset{C}{\underset{ij}{m}}-\sum_{k}\alpha_{k}\overset{S,k}{\underset{ij}{m}}\;\right)}^{\text{2}} \end{array}$$ (2)

Here, $\overset{k}{\underset{S}{M}}$ corresponds to the contact matrix of the k-th substructure, with their respective scaling factors $\alpha_{k}$. In the following the $\alpha_{k}$ values for the substructures are obtained by numerically minimizing the Equation 2 using Nelder-Mead algorithm implemented by the *Optim.jl* package [37] in Julia [38]. To assess the numerical stability of the solutions we run the minimisation procedure 10,000 times with random initial conditions followed by a k-means clustering implemented in the *Clustering.jl* package in Julia [39]. This allows us to identify the different local minima and estimate the global minimum. For the further analysis, we use the global minimum to define the contact numbers for the respective substructures in the ABM.

We compare the contact matrices for households, schools, workplaces, “other contact” and all contacts combined simulated in the ABM and based on survey data visually and quantitatively. For visual comparison, the differences between the simulated and survey-based contact matrices are displayed. Here, we show the differences in matrix form to be able to identify structural under- and overestimation of contacts between specific age groups. For quantitative comparison, the minimal SSE given by Equation 1 and 2, and mean relative difference given by:


$$\begin{array}{c} D\left(M_{C},\;M_{S}\right)=\frac{\text{1}}{\overset{\text{2}}{\underset{age}{N}}}\sum_{i,j}\frac{\left|\overset{C}{\underset{ij}{m}}-\overset{S}{\underset{ij}{m}}\right|}{\overset{C}{\underset{ij}{m}}+\overset{S}{\underset{ij}{m}}}, \end{array}$$ (3)

are used as measures of the difference between the two contact matrices. Here $N_{age}$ corresponds to the number of age groups which in our case is 17. We calculate the 95% confidence intervals (CIs) of the minimal SSE by bootstrapping with respect to the participants of the COVIMOD survey. The uncertainties of contact matrices estimated for the ABM are negligible due to the large sample size, which was ascertained by also bootstrapping with respect to the simulated individuals in GEMS, yielding CIs identical to the ones calculated by just bootstrapping the COVIMOD participants.

### Epidemic impact analysis

To assess the relevance of the identified differences between the contact matrices we formulate a simple age-structured SIR-model and analyse it using the next generation approach and numerical simulations.

For our model we use a simple age-structured SIR model with a static population given by:


$$\begin{array}{c} \frac{dS_{i}}{dt}=-\sum_{j}\beta m_{ij}\frac{S_{i}I_{j}}{N_{j}},\;\frac{dI_{i}}{dt}=\sum_{j}\beta m_{ij}\frac{S_{i}I_{j}}{N_{j}}-\gamma I_{i}, \end{array}$$ (4)

$$\begin{array}{c} \frac{dR_{i}}{dt}=\gamma I_{i}. \end{array}$$

Where, as before, i and j correspond to indices of the age group and $m_{ij}$ to the corresponding entries in the contact matrix. The model assumes age-independent recovery rates γ and infection likelihoods β.

The next generation approach is a common method of deriving the reproduction number based on an ordinary differential Equation-based model [40,41]. For the simple model SIR model given by Equation 4 the entries of next generation matrix $N_{ij}$ are determined by:


$$\begin{array}{c} N_{ij}=\frac{\beta m_{ij}}{\gamma }. \end{array}$$

Consequently, the reproduction number is equal to the spectral radius of the contact matrix, i.e., its maximum absolute eigenvalue, multiplied by pathogen specific values of beta and gamma:


$$\begin{array}{c} R_{\text{0}}=\text{max}\left|Eig(N)\right|=\frac{\beta }{\gamma }\text{max}\left|Eig(C)\right|. \end{array}$$

Since we are interested in the comparison of the survey-based and simulated contact matrices, we use the ratio of the two resulting reproduction numbers, thereby cancelling out the pathogen-specific parameters:


$$\begin{array}{c} \tilde{R_{\text{0}}}=\frac{\overset{C}{\underset{C}{R}}}{\overset{S}{\underset{\text{0}}{R}}}=\frac{\text{max}\left|Eig\left(M_{C}\right)\right|}{\text{max}\left|Eig\left(M_{S}\right)\right|} \end{array}$$ (5)

Here the indices C and S indicate the COVIMOD-based and simulated contact matrix, respectively.

Besides the reproduction number we are also interested into the spread of the pathogen on a longer time scale. For this we simulate our SIR model using the *DifferentialEquations.jl* package in Julia 1.10.4. Note that we are only interested in the comparison of the simulation outcomes between the two contact matrices such that the pathogen-specific parameters are arbitrarily set to γ=1/6 and β=0.08. The number of individuals in each age groups is determined from the census 2023 [23]. To incorporate the uncertainty of the COVIMOD contact matrix, we again bootstrap with respect to the COVIMOD participant, run the simulation for each sample, and aggregate the epidemiological quantities including their confidence intervals.

## Results

### Contacts in the setting “Household”

**Fig 1:**
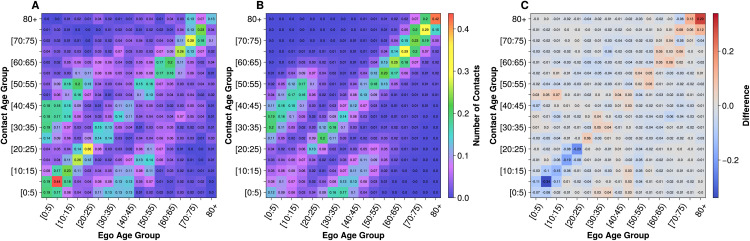
Comparison of the age x age contact matrices for household contacts. **(A)** Contact matrix for the household contacts derived from COVIMOD data. **(B)** Calibrated household contact matrix simulated in GEMS. **(C)** Difference between the GEMS and COVIMOD contact matrices (B – A) negative values correspond to an underrepresentation of contacts in GEMS compared to COVIMOD.

Fig 1 (A) displays the contact matrix for the household setting from the COVIMOD survey. The matrix shows a prominent diagonal line, corresponding to age-assortative mixing due to individuals of the same age living together as couples, siblings, or roommates. Additionally, two off-diagonal lines are present. These lines represent intergenerational contacts of families living together, such as the household contacts of children with their parents.

The calibrated household contact matrix generated based on the ABM population structure is shown in Fig 1 (B). It displays a similar pattern as the survey data, with a main diagonal representing age assortative living conditions and off-diagonal lines representing inter-generational contacts. In the ABM population structure-based matrix, the oldest age group (80+) shows the highest number of contacts within their own group, whereas in COVIMOD the children aged 5–10 exhibit the highest number of contacts. The difference is a result of the implemented form of random mixing, as it assumes the same number of daily contacts for all individuals. This causes individuals living in larger households with different age groups, such as families, to have a contact pattern distributed over the present age groups. At the same time, individuals living in small households with few or only one other individual have the same number of contacts as individuals living in families but with fewer different age groups.

Calibrating the contact sampling in the ABM with the COVIMOD contact matrix, by minimizing SSE between the contact matrices, results in an average number of household contacts of $\lambda_{H}=\text{0}.\text{9}\text{2}$ (no difference by household size is considered). The difference between the calibrated population structure-based contact matrix and the COVIMOD contact matrix is displayed in Fig 1 (C). The biggest differences arise for children and young adults under the age of 25. Here, the number of contacts people have with their own age group and neighbouring age groups is underestimated, where the largest deviations arise for the [5,10) age group. At the same time, the age assortative contacts of the 80 + age group are overrepresented. While the random contact sampling captures the overall structure of household contacts it fails to account for the high number of contacts among children and young adults, as well as the low social activity of the elderly.

### Contacts in the Setting “School”

**Fig 2:**
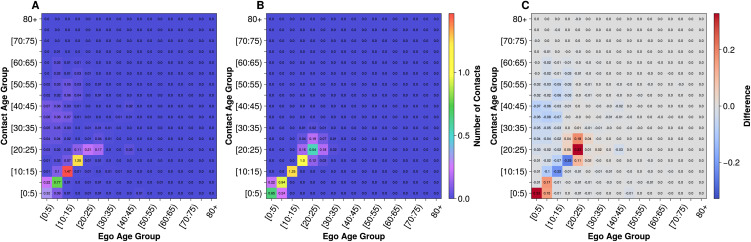
Comparison of the age x age contact matrices for school contacts. **(A)** Contact matrix derived from the COVIMOD survey data. **(B)** School contact matrix derived from the simulated contact behaviour in GEMS. **(C)** Difference between the GEMS and COVIMOD contact matrices (B – A) negative values correspond to an underrepresentation of contacts in GEMS compared to COVIMOD.

Fig 2 (A) shows the contact matrix for school contacts in the COVIMOD survey. Again, the age-assortative mixing is visible as a diagonal line. This mixing is facilitated by the structure of the school system, where children are in school classes with other children of similar age. The contact matrix also displays minor off-diagonal elements, which might stem from the choice of age group size for the youngest age groups. In Germany, children visit primary school from the age of six; therefore, contacts between the two youngest age groups may arise from kindergarten contacts between children under five years and children over five who have not yet been admitted to school. For the [20, 25) group the off-diagonal elements likely reflect contacts within universities, which exhibit a less age-dependent structure. Beyond the age of 25 the entries in the contact matrix vanish as people are likely to have left the educational system. Additionally, some contacts between young and middle-aged individuals can be observed reflecting contacts of students with teachers.

The contact matrices for all substructures of the school using the uncalibrated random contact sampling are displayed in Fig A in S1 Appendix. Here, the school classes and school years represent highly age-assortative mixing while schools and school complexes also capture contacts between age groups. Calibration of the average number of contacts for the substructures yields: $\lambda_{schoolyear}=\text{1}.\text{2}\text{6}$ and $\lambda_{class}=\lambda_{school}=\lambda_{schoolcomplex}\approx \text{0}$. As expected, the age structure of school years alone most closely replicates the contact structure observed in COVIMOD. Note that these average numbers of contacts correspond to the most common solution of the numerical minimisation. Using the k-means cluster analysis of the minimisation results with random initial conditions we identified this kind of solution as the global minimum being obtained 88% of times. The remaining minima correspond to local minima. Most common were combinations of school classes and school years, i.e., where $\lambda_{class}\neq \text{0}$ and $\lambda_{schoolyear}+\lambda_{class}\approx \text{1}.\text{2}\text{6}$. Less than 0.3% of solutions also show contributions of the school setting. Further information on the identified clusters is displayed in Table E in S1 Appendix. Looking at the values of Equation 2 for these solutions shows that $\lambda_{\text{schoolyear}}=\text{1}.\text{2}\text{6}$ is the global minimum, indicating that the small differences in the contact structures of the substructures leads to the best fit when only the schoolyear is included. In the following we use the contact numbers corresponding to this global minimum. The calibrated ABM school contact matrix is displayed in Fig 2 (B).

The difference between the calibrated ABM and the survey-based school contact matrices is displayed in Fig 2 (C). The random contact sampling in the ABM, substantially underestimates the age assortative contacts of the age group [10,15), while it overestimates those of the youngest age group. Besides the age-assortative contacts the mixing between neighbouring age groups is overestimated for the young and older age groups while it is underestimated for the groups in between, highlighting the limitation of the random contact sampling approach in this setting. Mitigating the under- and overestimation requires an age-dependent contact sampling to describe differences in social activity. Note, that a class-dependent social activity could also be defined as the class is directly related to the age. Additionally, the teacher-pupil contacts are not captured in the chosen ABM implementation due to the employment setting structure. Within the employment structure teachers are not included in the school setting but rather are treated as part of the regular workforce and assigned a workplace.

### Contacts in the Setting “Workplace”

**Fig 3:**
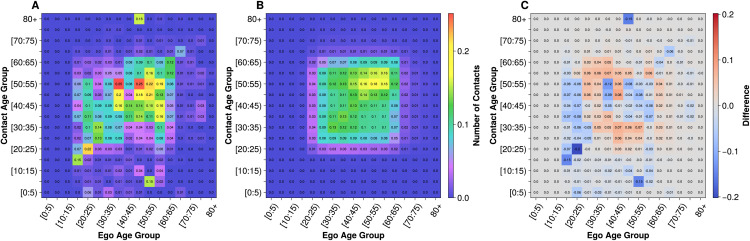
Comparison of the age x age contact matrices for workplace contacts. **(A)** Contact matrix for the COVIMOD workplace contacts including asymmetric contacts, e.g., teacher-pupil contacts. **(B)** Workplace contact matrix based on the contact behaviour simulated in GEMS. **(C)** Difference between the GEMS and COVIMOD workplace contact matrices (B – A) negative values correspond to an underrepresentation of contacts in GEMS compared to COVIMOD.

Fig 3 (A) displays the contact matrix for workplace contacts obtained from the COVIMOD survey. Workplace contacts emerge in the age group [15,20) and continue until the age group of [65,70) with only some contacts for older age groups, reflecting the typical retirement age of 65 years. Within this working population the number of contacts strongly fluctuates. Additionally, contacts of working individuals with elderly people and children can be observed, likely representing care providers and teachers, respectively.

The contact matrices for the four workplace substructures using the uncalibrated random contact sampling are presented in Fig B in S1 Appendix. As all substructures have a similar age structure, i.e., the age-constellation of workers in an office is on average identical to the age-constellation of entire companies, all substructures also exhibit a similar contact structure. The calibration of the contact numbers of the substructures with the COVIMOD data results in $\lambda_{workplace}=\text{0}.\text{2}\text{9}$, $\lambda_{department}=\text{0}.\text{8}\text{1}$ and $\;\lambda_{office}=\lambda_{workplacesite}\approx \text{0}$. As for the school contacts, we used k-means clustering to identify that this solution corresponds to the global minimum of the SSE. It was obtained in 91% of the runs. Most remaining solutions correspond to other combinations of workplace and department with $\lambda_{workplace}+\lambda_{department}\approx \text{1}.\text{1}$. In 3% of cases there are contributions from the office. Note, that the SSE differences between clusters displayed in Table F in S1 Appendix are much smaller for the workplace substructures than for the school substructures due to the similarity of the age composition and contact structures across substructures. In the following we will again use this global minimum for our analysis.

The calibrated ABM workplace contact matrix is displayed in Fig 3 (B). This matrix consists of a rectangular structure encompassing the working-age population starting from the age group [20,25) and ending with the [60,65) group, with a few contacts for the [65,70) group. The internal structure of the rectangle reflects the age distribution of the working population. Since the [50,55) group corresponds to the largest group in the working population, most contacts of all working ages occur with this group.

The difference in the working contacts between the contact survey and the population structure-informed ABM is shown in Fig 3 (C). It varies between over- and underestimation, due to the large variations in the COVIMOD data that can be observed in Fig 3 (A). A systematic underestimation of work contacts occurs for the [15,20) group, which only exhibits very few contacts in the ABM while COVIMOD shows a similar contribution as for older age groups. Additionally, asymmetric contacts, i.e., contacts between the working population and individuals outside this group, are underrepresented since the implementation of the ABM used for this study only includes working-age people in workplaces. Therefore, it is unable to represent these asymmetric contacts.

### Contacts in Other Settings

**Fig 4:**
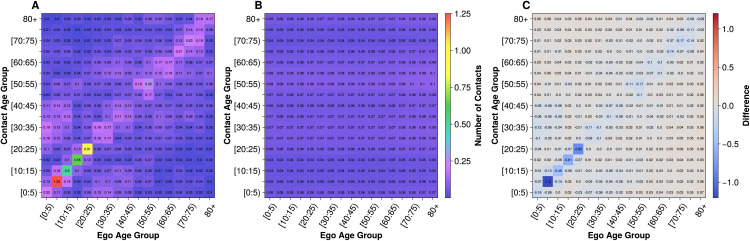
Comparison of the age x age contact matrices for the other contacts. **(A)** Contact matrix for the other contacts derived from the COVIMOD contact survey. **(B)** Contact matrix for other contacts based on the contact behaviour simulated in GEMS. **(C)** Difference between the GEMS and COVIMOD other contact matrices (B – A) negative values correspond to an underrepresentation of contacts in GEMS compared to COVIMOD.

Fig 4 (A) displays the COVIMOD contact matrix for the other contacts. The highest number of contacts occur along the diagonal for young adults, highlighting their age-assortative mixing. Additionally, off-diagonal elements are present, reflecting the interactions between parents and other children. The diagonal line exists for all age groups highlighting the age-assortative mixing of all age groups. However, especially middle-aged individuals show a higher contact diversity, interacting with a wider range of age groups. Contact structure becomes more age-assortative again with increasing age.

Fig 4 (B) displays the contact matrix obtained for the other contacts from the population structure of the ABM. In the analysed implementation, these contacts correspond to random mixing within the municipalities, resulting in the likelihood of a contact with an age group to be equal to the share of individuals in the municipality in this age group. Consequently, the observed contact structure corresponds to the overall age structure in the municipalities. This can be observed as horizontal lines in Fig 4 (B). Here, the random mixing fails to capture the age assortativity and heterogeneous social activity that the COVIMOD data displays. Calibrating the other contacts in the ABM to the COVIMOD data yields $\lambda_{\text{O}}=\text{1}.\text{1}\text{9}$.

The difference between the calibrated contact matrix and the COVIMOD matrix is shown in Fig 4 (C). The structure of the COVIMOD matrix is clearly visible in the difference matrix, highlighting the systematic underestimation of parts of the COVIMOD contact structure by the random contact sampling in this setting. Consequently, contacts outside of these age groups are slightly but systematically overestimated. This highlights the inherent structural limitation using random regional contacts to represent the age-structured other contacts observed in the COVIMOD data.

### All contacts

**Fig 5:**
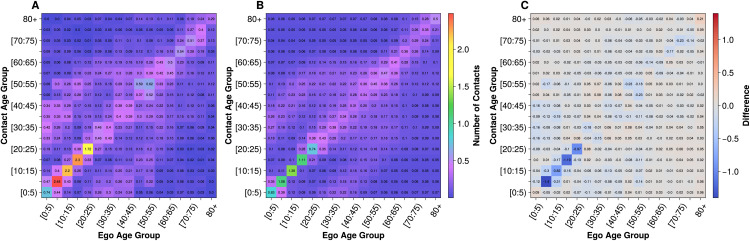
Comparison of the age x age contact matrices for all contacts combined. **(A)** Contact matrix for all contacts reported in the COVIMOD contact survey. **(B)** Combined contact matrix of all contacts simulated in GEMS. **(C)** Difference between the GEMS and COVIMOD contact matrices (B – A) for all combined contacts. Negative values correspond to an underrepresentation of contacts in GEMS compared to COVIMOD.

Fig 5 (A) and (B) show the contact matrices for all contacts observed in COVIMOD and simulated in the ABM, respectively. The difference between these matrices is shown in Fig 5 (C). While the random contact sampling in settings captures the overall contact structure visible in the survey data, it severely underestimates the age-assortative contacts of children and young adults as well as contacts between children and their parents. This difference is primarily due to the assumption of age-independent contact numbers and the limitations in modelling the “other contacts” mentioned before.

Table 1 presents the SSE and mean relative difference between the ABM population-generated and COVIMOD contact matrices for each contact setting calculated using Equation 1 and Equation 3, along with the 95% confidence intervals for the obtained through bootstrapping with respect to the COVIMOD participants. Household, school, and workplace contacts all show similar SSE values: 0.7 (95% CI: 0.4 to 0.9), 0.7 (0.3 to 1.1) and 0.5 (0.2 to 0.7), respectively. In contrast, the “other contacts” show a considerably higher SSE of 3.8 (1.2 to 6.5). Lastly, the deviations for all contacts lead to an SSE of 7.6 (95% CI: 2.3 to 13.0). The mean relative difference shows a trend opposite to the SSE, with the other contact and all combined contacts displaying a smaller mean relative difference with 0.29 (0.27 – 0.32) and 0.20 (0.18 – 0.22), respectively. Household, school, and workplace contacts all show differences around 0.4, which is higher but still in the same region as the other and all combined contacts. This reversed trend stems from larger numbers of average daily contacts between the age groups in the other and all combined contacts. Note, however, that for the spread of a pathogen relative differences are of lesser importance than absolute differences. At the same time, different transmission probabilities per setting, particularly with lower probabilities in other places would diminish the difference.

**Table 1:** Sum of squared errors (SSE) and mean relative difference between contact matrices in COVIMOD and the fitted GEMS calculated using Equation 1, with 95% confidence intervals determined by bootstrapping with respect to the COVIMOD participants.

Contact Setting	SSE (95% CI)	Relative Difference (95% CI)
Household	0.7 (0.4 - 0.9)	0.36 (0.34 – 0.38)
School	0.7 (0.3 – 1.1)	0.44 (0.42 – 0.46)
Workplace	0.5 (0.2 – 0.7)	0.45 (0.43 – 0.47)
Other Contacts	3.8 (1.2 – 6.5)	0.29 (0.27 – 0.32)
All Contacts	7.6 (2.3 – 13.0)	0.20 (0.18 – 0.22)

### Epidemic impact analysis based on a compartmental model

As described in the methods section, we compare the calibrated random mixing in settings contact matrix with the empirical contact matrix obtained from COVIMOD by incorporating both matrices into an age-structured SIR-model. Using the next generation matrix approach (Equation 5) we obtain a reproduction number ratio of ${\tilde{R}}_{\text{0}}=\text{1}.\text{3}\text{7}$, based on a spectral radius of 2.79 for the simulated contact matrix and 3.82 for the COVIMOD-based contact matrix.

Substantial differences also arise for the numerical simulations of the SIR-model using the COVIMOD-based and simulated contact matrices

and are apparent both in the overall epidemic progression and in the progression through the age groups. The SIR-model based on the COVIMOD contact matrix shows an earlier and more pronounced peak as expected given the larger reproduction number. The spread of the pathogen through the age groups displays a similar underlying dynamic. The initially infected individuals, i.e., the age group [20,25), spread the pathogen mostly to younger individuals between 5 and 20 years old. It spreads predominantly within this age group until the peak where the largest prevalence shifts towards older age groups. Compared to the COVIMOD contact matrix the simulated contact matrix results in spread that is less restricted to specific age groups. Even, during the initial phase, when for the COVIMOD contact matrix the pathogen almost exclusively spreads among the younger age groups the simulated contact matrix results in a high relative prevalence for adults and the elderly. Note, that we also observe this dynamic if other age groups are initially infected, as displayed in Fig I in S1 Appendix.

**Fig 6:**
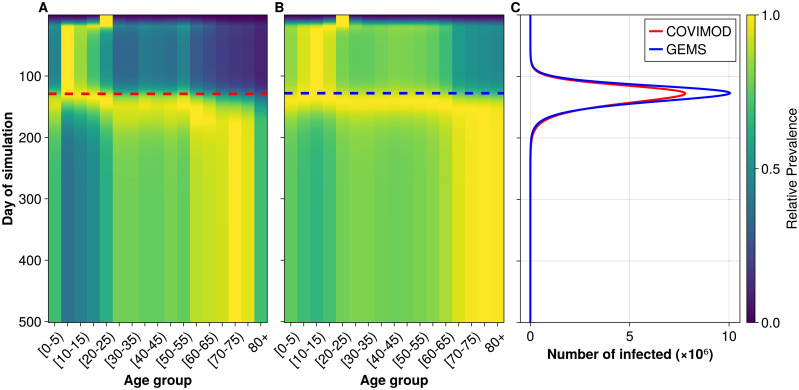
SIR model results using the modified beta for the GEMS-based simulation to achieve equal reproduction numbers. **(A)** and **(B)** display the relative prevalence in the age groups over the course of the simulation for the COVIMOD-based and GEMS-based contact matrix, respectively. **(C)** displays the number of infected over the course of the simulation.

**Fig 7:**
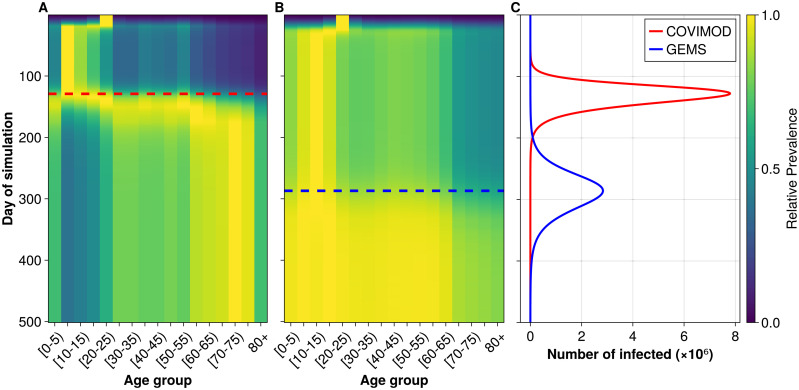
SIR model results. **(A)** and **(B)** display the relative prevalence in the age groups over the course of the simulation for the COVIMOD-based and GEMS-based contact matrix, respectively. **(C)** displays the number of infected over the course of the simulation.

As these observations are also related to the difference in reproduction number, we use the prior ratio ${\tilde{R}}_{\text{0}}=\text{1}.\text{3}\text{7}$, derived from the next generation matrix to modify beta for the contact matrix simulation, i.e., $\beta_{S}={\tilde{R}}_{\text{0}}\;\beta$. We display the comparison in Fig 6, where the panels are analogous to Fig 7. The applied adaptation of beta makes the similarity of the two simulations more apparent. In both simulations the pathogen first spreads within the initially infected age group after which it spreads mostly among children and only once the peak is reached the spread shifts towards older age groups. While basic dynamics in both simulations are similar, the simulation based on the simulated contact matrix remains less restrictive concerning the spread across age groups which lead to a broader spectrum of age groups being involved in all phases of the simulation. Lastly, one should note that the simulation based on the simulated contact matrix yields a higher peak number of infected individuals.

In Table 2 we compare key metrics of disease spread between simulations based on the COVIMOD and ABM contact matrices for both the initial parametrisation and modification of beta for the ABM-based simulation. All differences were calculated by subtracting the variable for the simulated contact matrix from the variable for the COVIMOD-based contact matrix such that negative values indicate higher values for the simulated contact matrix. The results show that while the initial parametrisation leads to a faster dynamic with more infections for the COVIMOD-based contact matrix, aligning the reproduction numbers by using a modified beta leads to a higher number of infections for the simulated contact matrix while the day of peak infections is the same for both matrices.

**Table 2:** Differences in epidemic outcomes between the COVIMOD-based and simulated contact matrices for the initial parametrisation and the modified beta for the simulated contact matrix. Negative values indicate higher outcome values of the simulated contact matrix.

Outcome Difference	Initial Parametrisation (95% CI)	Modified Beta (95% CI)
Attack Rate	0.17 (0.13; 0.21)	-0.11(-0.15; -0.07)
Peak Infections (10^6)	5.0 (3.8; 6.3)	-2.2(-3.4; -0.9)
Peak Day	-160 (-190; -140)	-2.2(-27.5;19.0)
Final Susceptible (10^6)	-15 (-18; -11)	9.3(5.8; 12.8)

## Discussion

In this study, we assessed if random mixing in settings (as the implementation of a population structure-triggered contact behaviour in ABMs) could accurately represent contact behaviour as measured through contact matrices.

Our results demonstrate that the implemented contact sampling strategy captures the basic structure of empirically assessed contact behaviour in settings with a restricted age-structure such as schools and workplaces. Specifically, random sampling can represent the age assortative contacts in schools and households, the children-parent interactions in households, as well as the homogeneous mixing of the working population for workplace contacts. However, all these contacts show small deviations from the contacts observed in contact surveys with respect to two dimensions. First, the implemented random contact sampling assumes the same average number of daily contacts for all individuals in the same setting, which fails to account for heterogeneous social activity known to be age-dependent [42]. Thus, mismatches arise, especially for children and the elderly. The largest differences arise for the setting of other contacts

This is a result of the chosen implementation of the ABM, which models “other contacts” as random contacts in the municipality. This approach fails to account for the heterogeneous contact behaviour observed in COVIMOD. While the trend is less pronounced when considering group contacts, it is still prevalent. Looking at the relative differences between the contact matrices derived from COVIMOD and the population structure of the ABM reveals an opposing trend with all differences being of the same order of magnitude but with “other contacts” showing the smallest differences. This opposite trend is a result of the high number of daily contacts in the other category. It highlights the importance of correctly representing other contacts since even as they are incorporated relatively well the SSE still shows the largest deviations for these other contacts. At the same time, the contacts in other settings could play a smaller role epidemiologically due to lower transmission probabilities during a contact.

The comparison of the resulting contact matrices using an age-structured SIR-model indicates that the differences in the contact matrices could translate into substantial differences in the epidemic outcomes. Using the next-generation matrix approach resulted in a reproduction number ratio substantially larger than one, corresponding to a faster disease spread when using the survey-based compared to the simulated contact matrix in the SIR-model. We also observed differences of the disease dynamics simulated in the SIR-model, including differences in outcomes such as the final attack rate and, peak infection numbers. In addition to these aggregate epidemiological indicators, the two contact matrices produced broadly similar but still noticeably different spreading dynamics between age groups. These differences became more apparent when both simulations were adjusted to equal reproduction numbers. We obtain a similar impact on the infectious disease simulation when we include group contacts.

Together these findings suggest that simplified algorithms for contact behaviour based only on population structure in ABMs may lead to unintended consequences when modelling infectious diseases. However, our comparison is based on a simple SIR model, which does not take into account factors such as contact clustering or repeated interactions which influence transmission dynamics both in the real-world and in ABMs. Therefore, while integrating both contact matrices into the SIR model yields different spreading dynamics and epidemic outcomes, estimating differences between real-world spreading dynamics and those in an ABM warrants further investigation.

The discrepancies between the contact structures implied by random mixing within settings assumptions in an ABM and contact surveys could be reduced by employing more refined contact sampling methods. Potential approaches include a setting-size dependent number of contacts. For households, the correlation of increasing numbers of contacts with increasing household size has been demonstrated in [20]. However, it is not obvious to which extent the underestimation of child-child contacts is a result of the lack of household size dependence or actually a result of a higher number of contacts of children with their siblings. The latter has also been shown to be a determining factor for household contacts [43].

The inclusion of a setting-size-dependent number of contacts can be understood as the inclusion of more setting types with their own average number of contacts, i.e., households with a specific size (range) would correspond to a new setting type. The inclusion of further settings could be a potential solution to reduce differences between contact patterns in the real-world and in ABMs. Extending the implemented settings to include additional settings such as supermarkets or representing the existing settings on a more granular scale, for example, by replacing the large municipality settings with local communities [44]. This more granular scale could ensure that the arising contacts more closely represent the contact structure of the other contacts in COVIMOD. However, this approach requires additional data on the members of such settings which is rarely available. Otherwise setting association must be based on assumptions made by the modellers. While such approaches could introduce more granular settings they would continue to be based on homogeneous mixing within these settings.

The representation of heterogeneous contact behaviour requires methods that include household size dependent number of contacts and contact probabilities dependent on attributes of the potential contacts, such as their age. Similar attribute-based approaches could address the limitations in representing the other contacts. However, such attribute-based approaches would drastically increase the model’s computational complexity, as the probability of sampling an individual as a contact would depend on the properties of both contact partners. One simplified solution could be the implementation of two independent subsettings of which one captures age assortitiveness while the other does not.

Representing heterogeneous other contacts could also be achieved while maintaining the low computational complexity of random mixing within settings. Assuming a strong overlap between the other contacts and the contacts occurring in schools, workplaces or households a potential approach involves sampling additional contacts within these existing settings to represent the heterogeneous mixing observed in COVIMOD. Using this approach for the structure observed for the “other contacts” together with the currently used random municipality-based contacts could represent both random and structured other contacts.

Limitations of this study arise mainly from the choice of the data sources feeding into the comparison. While contact surveys such as COVIMOD are the main workhorse for studying individuals’ contact behaviour in the context of infectious diseases, they exhibit inherent challenges [45]. In particular, selective memory likely leads to the underreporting of random contacts in COVIMOD, i.e., chance encounters with individuals that are not known to the participant such as contacts in public transport [2,46]. Other contacts in GEMS, implemented as random regional contacts, might correspond to those random contacts underrepresented in COVIMOD, since these are also chance encounters in the regional population. This could to some degree explain the observed difference.

Furthermore, in COVIMOD, parents reported on the contacts of their children as proxies, which limits the validity of their reported contact behaviour, especially for school contacts [47]. In addition, since some of the parents were asked to fill in the questionnaire on behalf of their children and not for themselves parents are slightly underrepresented in the survey data as respondents for the adult group. For the household and other contacts, the symmetry condition was applied to mitigate this shortcoming. Another limitation arises from treating participants who contributed to multiple survey waves as independent observations. As approximately 2,200 participants took part in more than one survey, treating them as independent will introduce bias. This includes a reduction in the effective sample size and an underestimation of uncertainty due to more stable, repeated individual contact patterns assuming that participants retain some of their contact behaviour between survey waves. However, since our analysis focuses on population-level contact behaviour rather than individual-level inference, we consider this bias to be acceptable for the purposes of this study.

Further limitations arise from the group contacts reported by individuals. While including this option in COVIMOD allows the participants to easily record a large number of contacts with little effort, potentially leading to more accurate information on the number of contacts, it only includes little information on the age of contact partners. We addressed this limitation by excluding group contacts from our main analysis to only investigate the structure of contacts where the age group is known. We support this analysis by using two different imputation strategies to assess the impact of including group contacts. From this additional analysis we draw the same conclusions about the random mixing in settings assumption. However, we observe that the effect size depends on the inclusion/exclusion of group contacts and the method employed for imputing the age of contact partners.

As an abstraction of reality, the population structure-triggered contact behaviour in the ABM introduces further limitations arising from the Gesyland population data and the models’ set-up. As the contact structures that emerge based on random mixing in settings are mainly dependent on the correct association of individuals to settings, the Gesyland population itself constitutes the main limitation. The assessment of the random mixing within settings relies on accurately defined settings that capture the real (age) constellation of settings such as household compositions. While we assume the accurate association of individuals to settings the differences of the simulated and empirical contact structures could also be partly caused by inaccurate association of individuals to settings within Gesyland. Gesyland contains various approximations of the German population, including identical occupations within workplaces. Due to limited data, characteristics that occur in less than 1% of the population are omitted, and care units (e.g., nursing homes) and institutional units (e.g., prisons) are generated based on educated guesses. Both of these are treated as households in the ABM. Additionally, the implemented ABM structure strictly separates settings by types and the roles individuals associated to them fulfil, e.g., only students are assigned to school while teachers are assigned to workplaces. Therefore, only individuals in the same “role” can be in contact. As a result, the chosen implementation of the ABM is unable to represent asymmetric contacts such as student-teacher contacts regardless of the used contact sampling method. However, ABMs in general commonly only include the symmetric contacts, making the chosen implementation a suitable model to evaluate the challenges of population structure-informed contact behaviour in ABMs.

As described above, the epidemic impact analysis performed has to be interpreted with caution. While we identify mismatches of the transmission dynamics when parametrising an SIR model with the COVIMOD-based vs the simulated contact matrix, these mismatches cannot be directly transferred to mismatches between real-world infections and infections simulated in the “full” ABM. Within our analysis we use the SIR model as an approximation of both the real-world and the ABM. This approach implicitly assumes that contact matrices alone suffice to represent infections and neglects features such as the clustering of contacts which we know to influence real-world and simulated disease dynamics. Furthermore, we assume that COVIMOD with its previously discussed limitations accurately captures all contacts relevant for disease transmission. We know that these assumptions are only approximations and do not hold exactly. Consequently, our results should be viewed as an indication that mismatches might exist. Further studies should accurately assess this indication by simulating different contact behaviours within the ABM to identify their potential impact on disease transmission.

Future research should refine contact sampling methods in ABMs to better represent the contact behaviour observed in the real-world, especially for studies focusing on disease dynamics in specific subgroups. Other types of contact data, such as sensor data, could be helpful for the calibration of contact behaviour. Sensor data, which is collected through devices that automatically record contacts based on proximity to other devices have been shown to record chance encounters more reliably compared to classical survey data, making it more suitable to parametrise random contacts [45,48].

Besides the average number of contacts, which was the focus of this study, the distribution of the number of contacts, i.e., inter-individual heterogeneity is crucial for the spread of infectious diseases and should be studied using data such as the COVIMOD survey [49]. In addition, the intra-individual heterogeneity, i.e., the daily variability in an individual’s contacts is another relevant factor for disease spread [50–52]. This heterogeneity could be represented in the implemented ABM version by using the existing substructure levels. Sampling the contacts in large substructures such as the school complex rather than the school class increases the heterogeneity of contacts as contact partners are likely to vary daily. Combining contacts in different substructures allows modelling different levels of intra-individual heterogeneity.

Although this study focuses on representing contact behaviour in ABMs, random mixing assumptions are also employed to construct contact matrices or networks [18–22]. These approaches generate synthetic populations and settings using population-specific datasets, such as census data, and define mixing behaviour within these settings. Based on these populations, age-stratified contact matrices or networks of the synthetic population are obtained. While some of these approaches also use a random mixing assumption, others incorporate more complex mixing behaviours, which could inform the development of ABMs. However, in ABMs contacts must be sampled at each time step, which requires efficient sampling methods. In contrast, contact matrices and networks are constructed once, corresponding to a single time step for the ABM. Nevertheless, these similar approaches may provide further insight into more refined contact sampling methods and help define metrics for comparing contact structures.

While the suggested refinements may allow for a more detailed representation of contact behaviour in ABMs, their impact on the simulated disease spread within the ABMs remains an open question.

Whenever contact behaviour is simulated, the implications need to be reviewed. A comparison of the simulated contact behaviour with real-world data, following the methodology presented in this study, should be integrated into the development process of any ABM to identify potential problems triggered by the implicit contact behaviour simulation.

## Supporting information

S1 Appendix The section “Contact Settings” in S1 Appendix includes descriptions of the contact settings implemented in GEMS, including household, school, workplace, and other contacts. The uncalibrated contact matrices for the hierarchical substructures of schools and workplaces are displayed Fig A in S1 Appendix and Fig B in S1 Appendix, respectively. The section “Group Contacts” includes the analyses identical to the main text but using two age-inference approaches for the age groups of group contacts (population age distribution–based and contact matrix–based inference), which were excluded in the main text. Clustering results from numerical minimisation procedures for school and workplace contacts are presented in the section “Clustering Results” (Table E in S1 Appendix and Table F in S1 Appendix). In section “Epidemic Impact” of S1 Appendix we assess the effect of the initially infected age group on disease transmissions in our age-structured SIR model (Fig I in S1 Appendix).
(DOCX)
