# Supplementary material for: A comparison of contact patterns derived from the population structure in agent-based models and empirical contact survey data
Source: PLoS Comput Biol. 2026 Jun 18;22(6):e1013533. doi: 10.1371/journal.pcbi.1013533 (PMC13309034; doi:10.1371/journal.pcbi.1013533)
Supplement: S1 Appendix — The uncalibrated contact matrices for the hierarchical substructures of schools and workplaces are displayed Fig A in S1 Appendix and Fig B in S1 Appendix, respectively. The section “Group Contacts” includes the analyses identical to the main text but using two age-inference approaches for the age groups of group contacts (population age distribution–based and contact matrix–based inference), which were excluded in the main text. Clustering results from numerical minimisation procedures for school and workplace contacts are presented in the section “Clustering Results” (Table E in S1 Appendix and Table F in S1 Appendix). In section “Epidemic Impact” of S1 Appendix we assess the effect of the initially infected age group on disease transmissions in our age-structured SIR model (Fig I in S1 Appendix). (DOCX) [file pcbi.1013533.s001.docx]

# Supporting Information

## Contact Settings

Households are one of the most important contact sites for the spread of many infectious diseases, as household contacts are generally close contacts that often involving physical contacts, and occur over long periods of time [53,54]. Compared to other types of contacts, household contacts are difficult to avoid because the shared living space can rarely be completely separated. Therefore, especially during periods of severe contact restriction, such as lockdowns, household contacts are critical to the pandemic’s progression. This highlights the need for models to accurately reflect the household contact structures, especially for models that aim to investigate the effects of contact restrictions such as GEMS.

In GEMS households are determined by the synthetic population generation of Gesyland and are modelled without any substructure assuming a random mixing between all household members. Using the uncalibrated model parameter $\lambda_{H}=1$ all individuals living in households with more than two members have on average one contact per day, while single households have none. This causes the average number of contacts in the household to be less than $\lambda_{H}$. Since the contacts of individuals living alone are not affected by varying $\lambda_{H}$ the average number of contacts still scales linearly with $\lambda_{H}$ such that the calibration can still be applied.

Schools were subject to substantial restrictions in many countries. These restrictions were not enforced without reason as schools have been identified as significant contributors to the spread of pathogens [55]. Accurately representing the contact structure without restrictions is key to understanding how restrictions in schools can mitigate pathogen spread. Contacts in the school include both close-proximity indoor contacts and physical contacts ideal for the transmission of various pathogens. The age-structure of these school contacts in Germany is directly related to the class system, as most contacts occur in the school classes where only students of a similar age meet. However, during school breaks there may also be contacts between individuals of different classes and ages leading to a higher level of contact heterogeneity.

In GEMS the school is comprised of four substructures that include the school classes, school years, schools, and school complexes. Here, the latter can always include multiple ones of the previous. This structure mimics the German school system, in which multiple schools frequently share a common schoolyard or other facilities, leading to interactions between students from different schools. Within schools, contacts between different schoolyears are facilitated, as the school years are often allocated to the same building. Contacts in the schoolyears arise independently of the class structure, e.g., through friendships that occur between different classes or lessons that include students from multiple classes. However, most contacts occur between individuals from the same class, as students spent a majority of their time in the same room as their classmates and form social groups in those classes. The structure of GEMS allows to capture all these contacts and specify their frequency individually. As in the COVIMOD data schools in GEMS also include kindergartens and universities.

Workplaces are locations where most adults spend a large proportion of their waking hours and have a large proportion of their daily contacts. Like children’s school contacts, workplace contacts often take place over long periods of time, e.g., in shared office spaces and occur during different activities such as talking or eating together. The structure of workplaces is highly heterogeneous resulting in very heterogeneous contact behaviours. While some people are self-employed and work alone, others work in shared office spaces with shared cafeterias. The daily contact behaviours of these individuals differ significantly. Similarly, the contacts at work might be stable as in the case of office workers or vary from day to day as in the case of restaurant workers or clerks. Another influencing factor is remote working, which results in a complete physical separation of the people while still maintaining productivity. However, this is not possible for all types of work.

In GEMS, workplaces, like schools, have four substructures. They consist of offices, departments, workplaces and workplace sites. Again, the latter includes at least one of the previous. The age structure in all substructures of the workplaces is similar, with no age-assortivity. While the substructures have little effect on the age groups that come into contact with each other they do contain different pools of people that allow for different levels of mixing. Inidividuals who work in the same office will have more contacts with each other than individuals who work in the same workplace site (building) but have no other work relations. However, the pool of potential contacts is much larger for the workplace site leading to a more homogeneous mixing of the population. Therefore, the main aim of using the substructures for workplaces is to be able to represent different levels of mixing of the population.

Other contacts in this analysis are all contacts that occur outside of the household, school, and workplace. These contacts include a wide variety of interactions, such as random contacts in shops but also contacts with friends and family outside of the household. These types of contacts were the main targets of contact restrictions during the Covid-19 pandemic. However, it is almost impossible to completely ban these other contacts, as they include contacts that arise during essential activities, such as going to the supermarket or to the doctor. Therefore, an accurate parametrisation of other contacts is essential to model the disease spread with and without restrictions.

While the COVIMOD dataset includes further distinctions of the contact type, such as shopping activities or sports, we have aggregated these types into other contacts to allow the comparison with the contacts generated in GEMS. GEMS uses an individual’s municipality as the location for other contacts. This can represent random contacts between individuals in supermarkets or waiting rooms but cannot capture reoccurring contacts such as regular meetings with friends. This approximation is used to maintain the static association of individuals with settings while also keeping the number of settings small enough to be efficiently handled.

### Substructure Contact Matrices


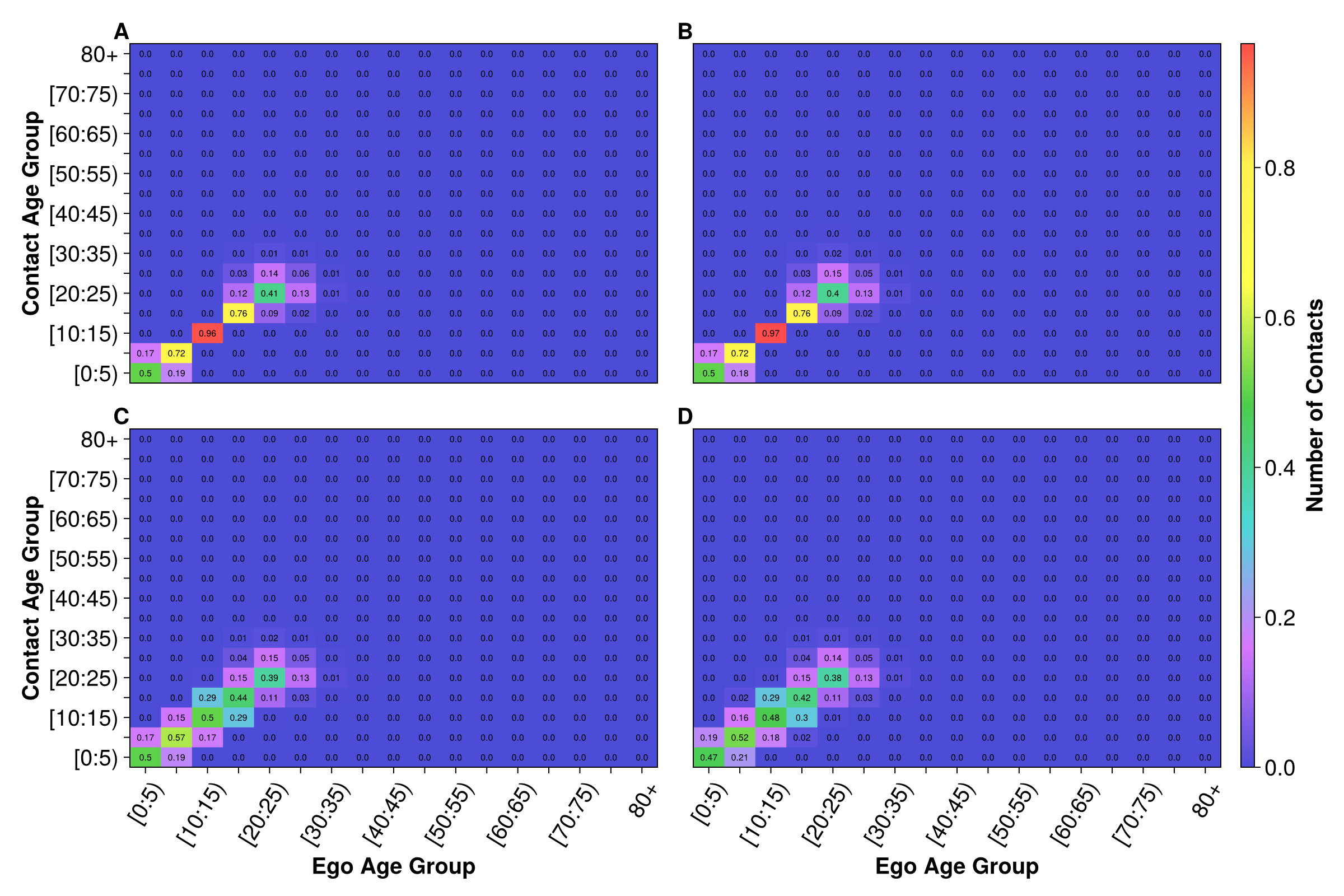


Fig A: Age x age contact matrices for all hierarchical levels of the school including the (A) school class, (B) school year, (C) school and (D) school complex in GEMS. Note that the school classes and years as well as the school complex and school exhibit have similar age structure, respectively such that the contact structures are almost identical.


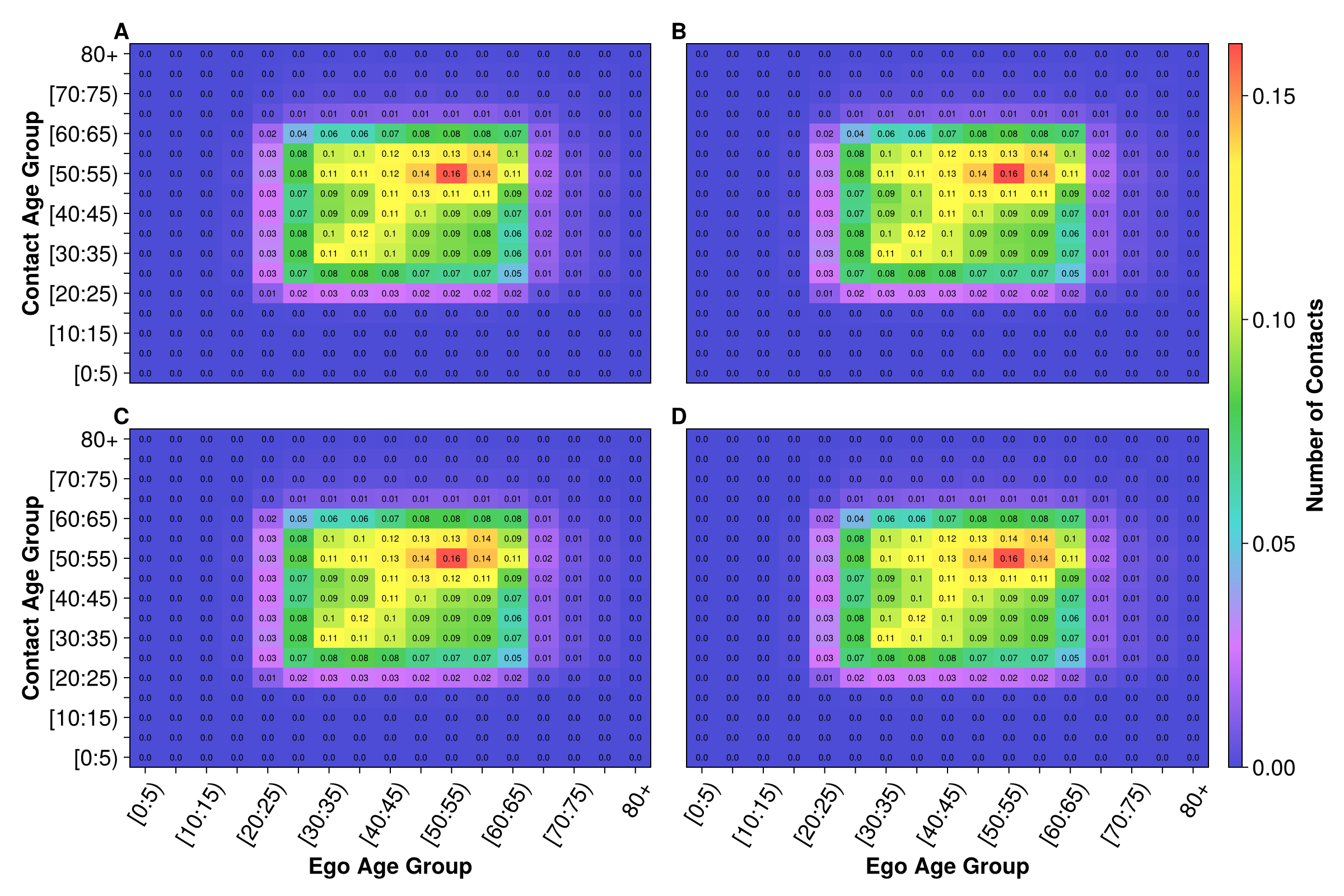


Fig B: Age x age contact matrices for all hierarchical levels of the workplace including the (A) office, (B) department, (C) workplace and (D) workplacesite in GEMS. Note that the age structures of all hierarchical levels of the workplaces are similar, such that the contact structures are almost identical.

## Group Contacts

Within our work we considered the COVIMOD contact patterns of all contacts where individuals reported the specific age group of these individuals in five-year age groups. In addition to these contacts, participants were able to report their group contacts. For these contacts, participants had to provide a single age group for all individuals that participated in this group contact (0-18, 18-65 or 65+) as well as the number of people they have had the group contact with.

While we excluded the group contacts in our main text, due to the difficulties in identifying the actual age groups of the group contact partners and thus the limited comparability with the simulation derived contact matrices, we analyse the contact survey data with group contacts in the following. Including group contacts leads to a total of 38,864 contacts including 8051 household contacts (with 0% group contacts), 10021 work contacts (54.4% group contacts), 5425 school contacts (56.3% group contacts) and 15,366 other contacts (33.8% group contacts). We employ two different methods to infer the age of contact partners from age groups provided by participants. First, we consider sampling the age from the age-distribution of the German population within the provided age group. Second, we use the contact matrices derived without group contacts to sample the age-distribution from the contact matrix. The corresponding results are presented in the following.

### Age-inference based on population age distribution

Within this section we compare the contact structure between the ABM-based contact matrix and the COVIMOD contact matrix including group contacts. Here, we determined the age of the contacts by drawing from the age-distribution of the German population, derived from the German census 2023 [23], restricted to the age group provided for the group contact by the participant. Thus, for a group contact of 50 contact partners in the age group 18-65 we sampled 50 ages between 18 and 65 with likelihoods proportional to the number of people in Germany of each age. Note that this imputation approach corresponds to the assumption of a randomly mixing population with the restriction of age groups.

Fig C displays the COVIMOD contact matrix (left column (1)), the simulation-based contact matrix (middle column (2)), and the difference matrix (right column (3)) for all settings. Note that we use the same colour scaling for all settings. Comparing the displayed contact matrices for the COVIMOD survey to the contact matrices without group contacts it is apparent that the mixing behaviour is less age assortative. Especially schools show a higher contribution of contacts in neighbouring age groups. The reason for these changes is the inference of group contacts based only on the wide age groups provided by the participants leading to a less age assortative behaviour.

The calibration of the setting-specific number of contacts leads to $\lambda_{H}=0.93$ for the household contacts, to $\lambda_{\text{department}}=\lambda_{\text{workplace}}=\lambda_{\text{workplacesite}}\approx0$, $\lambda_{\text{office}}=1.99$ for the workplace, $\lambda_{\text{class}}=\lambda_{\text{school}}\approx0$, $\lambda_{\text{schoolcomplex}}=0.99$, $\lambda_{\text{schoolyear}}=1.10$ for school contacts and $\lambda_{\text{O}}=1.80$ for other contacts. Similar to the results without group contacts we report the global minima for the substructures other combinations are possible but lead to local minima. Compared to the analysis without group contacts, we now get a contribution from the less age-assortative school complex setting that includes pupils of different ages. This can be attributed to the less age-assortative mixing arising from the group contact age inference.
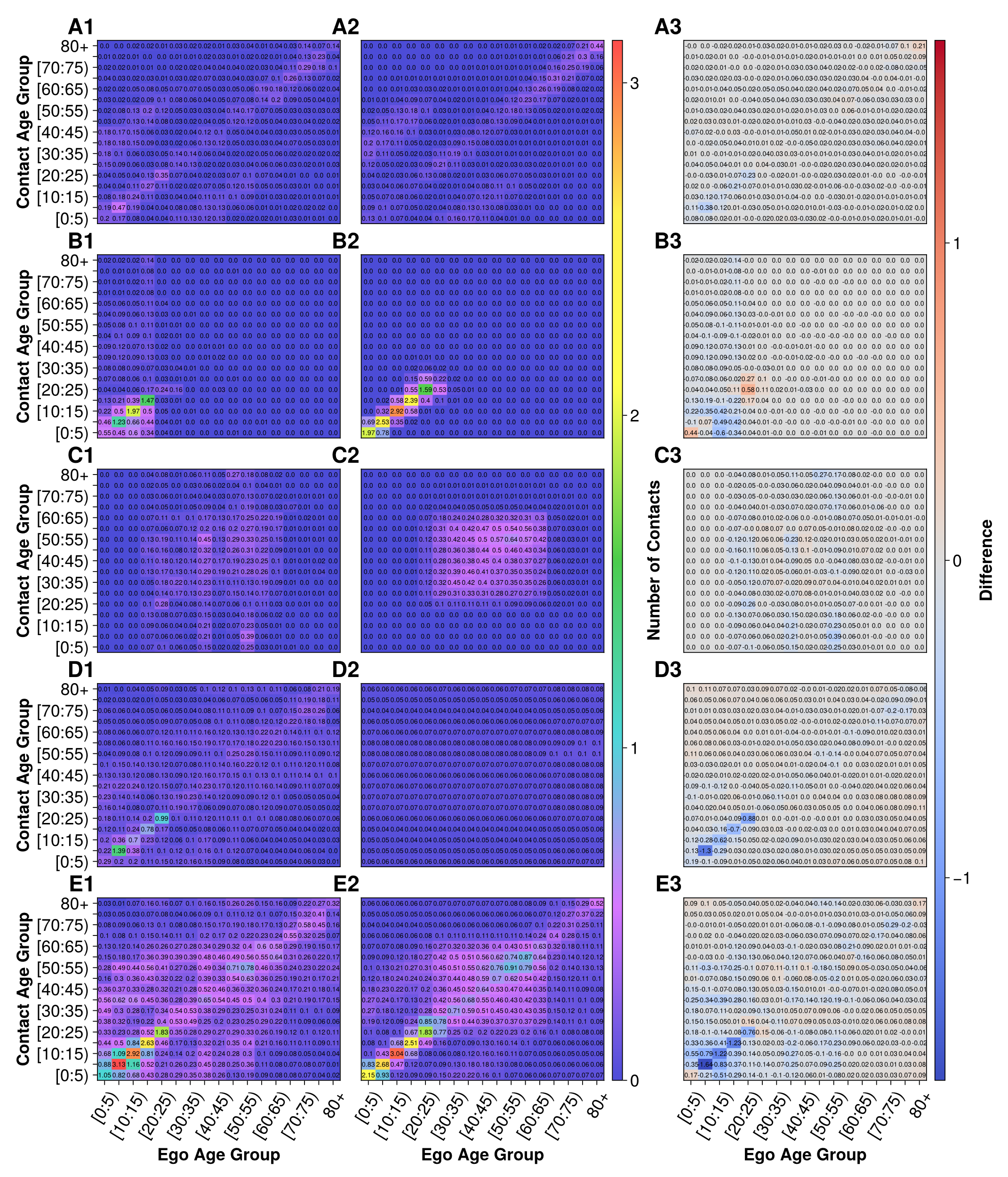


Fig C: Comparison of the age x age contact matrices for all contact settings using group contacts ages inferred by the populations age distribution. Each row corresponds to one contact setting, specifically they display (A) household, (B) school, (C) workplace, (D) other and (E) all contacts. The columns correspond to the (1) Contact matrix for the respective contacts derived from the COVIMOD contact survey, (2) Contact matrix for respective contacts based on the contact behaviour simulated in GEMS, and (3) difference between the COVIMOD and GEMS-based contact matrices for the respective contact setting.

The SSE and mean relative differences for the different settings are displayed in Table A. Here, trends similar to those observed for the analysis without group contacts can be observed. The other contacts continue to have the highest SSE. However, all SSE values have increased compared to the analysis without group contacts although by varying factors. The highest increase can be observed for the school contacts (4-fold) followed by the workplace contacts (3.6-fold) and the other contacts (1.4-fold). The smaller increase of the SSE for the other contacts is probably a result of the group contacts sampling based on the age distribution. The other contacts in GEMS correspond to a random mixing of all age groups, resulting in contact likelihoods proportional to the age-distribution of the population. Using also an age inference procedure that is also proportional to the age-distribution of the population will inevitably lead to a better matching between the two contact structures.

Table A: Sum of squared errors (SSE) and mean relative difference between contact matrices in COVIMOD including group contacts inferred by the populations age distribution and the fitted GEMS calculated using Equation 1, with 95% confidence intervals determined by bootstrapping with respect to the COVIMOD participants.

| **Contact Setting** | **SSE (95% CI)** | **Relative Difference (95% CI)** |
| --- | --- | --- |
| Household | 0.7 (0.4 – 1.0) | 0.37 (0.35 – 0.39) |
| School | 2.8 (1.6 – 3.9) | 0.51 (0.49 – 0.54) |
| Workplace | 1.8 (0.4 – 3.1) | 0.46 (0.44 – 0.48) |
| Other Contacts | 5.0 (2.0 – 8.0) | 0.25 (0.23 – 0.28) |
| All Contacts | 13.5 (7.6 – 19.4) | 0.19 (0.18 – 0.22) |

#### Epidemic Impact Analysis

To assess the epidemic consequences of the different contact matrices we again employ the age-stratified SIR model given by Equation 4. Calculating the reproduction number ratio using the spectral radii of 6.21 for the COVIMOD-based matrix and 4.14 for the simulation-based matrix with Equation 4 yields $\tilde{R}_{0}=1.50$. Thus, including group contacts and sampling their age from the populations age distribution leads to an even larger ratio of reproduction numbers and thus a more pronounced difference in the infectious disease dynamics. Note, that due to the increased number of overall contacts the dominant eigenvalues of both matrices have increased.

Fig D and Fig E display the relative prevalence in the individual age groups over time for the equal disease parametrisation and adapted beta value for the GEMS-based contact matrix, respectively. Similar to our prior analysis we observe a similar spread through the age groups for both contact matrices. However, the spread for the GEMS-based contact matrix is again less restricted to specific age groups and spread through more age groups for the entire simulation. The modification of beta for the GEMS-based simulation using the ratio of reproduction numbers has a similar effect as before and the similar spreading behaviour through the age groups becomes more apparent. Before the peak, the infections mainly occur in the younger age groups which changes after the peak, where the prevalence is highest in the older age groups. During the entire simulation, the prevalence is spread more equally over the age groups in the case of the GEMS-based contact matrix. Compared to the case without group contacts both simulations show a more evenly spread prevalence caused by the less age-assortative mixing.


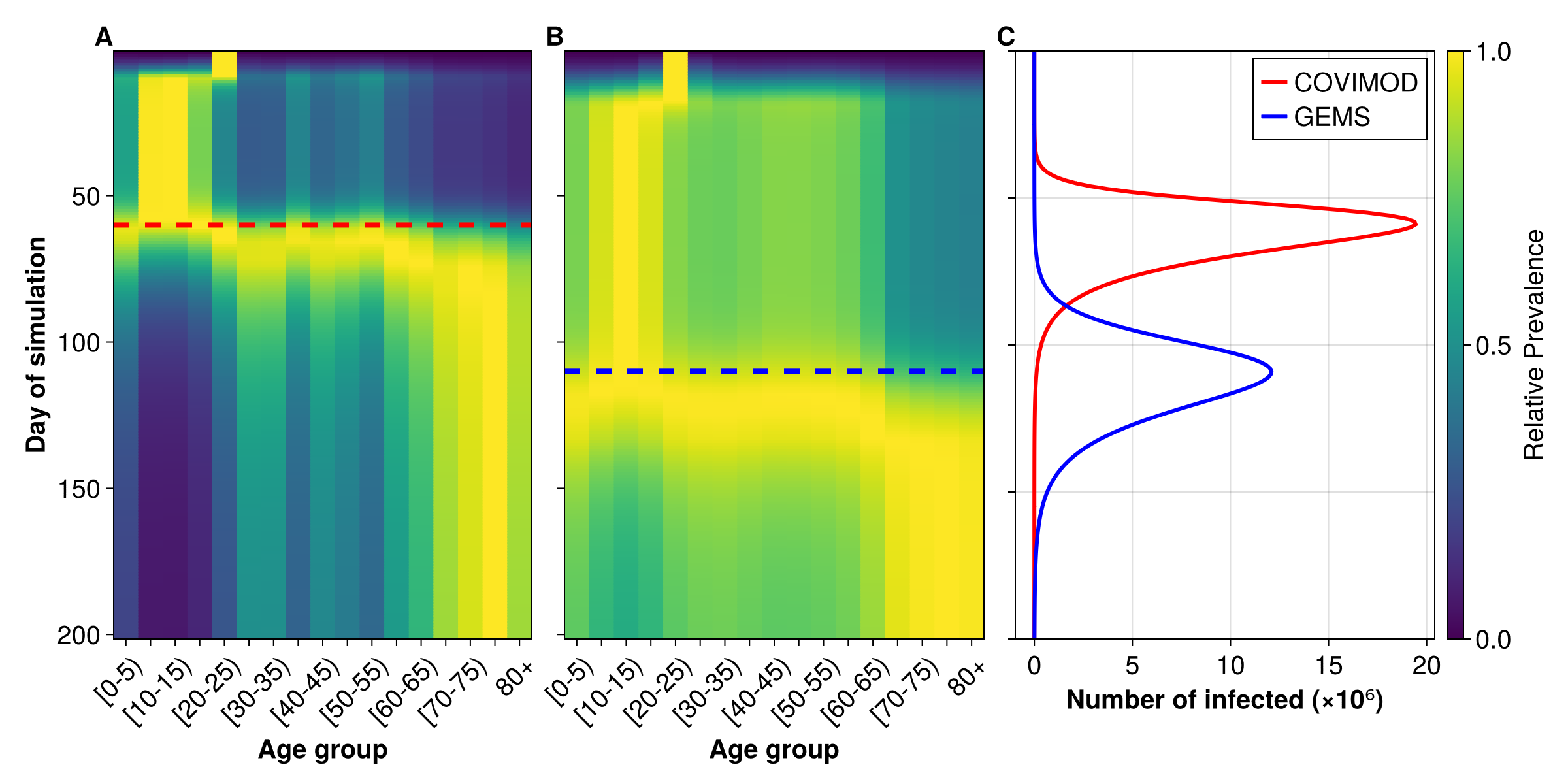


Fig D: SIR model results for group contacts derived from the populations age-distribution. (A) and (B) display the relative prevalence in the age groups over the course of the simulation for the COVIMOD-based and simulated contact matrix, respectively. (C) displays the number of infected over the course of the simulation.


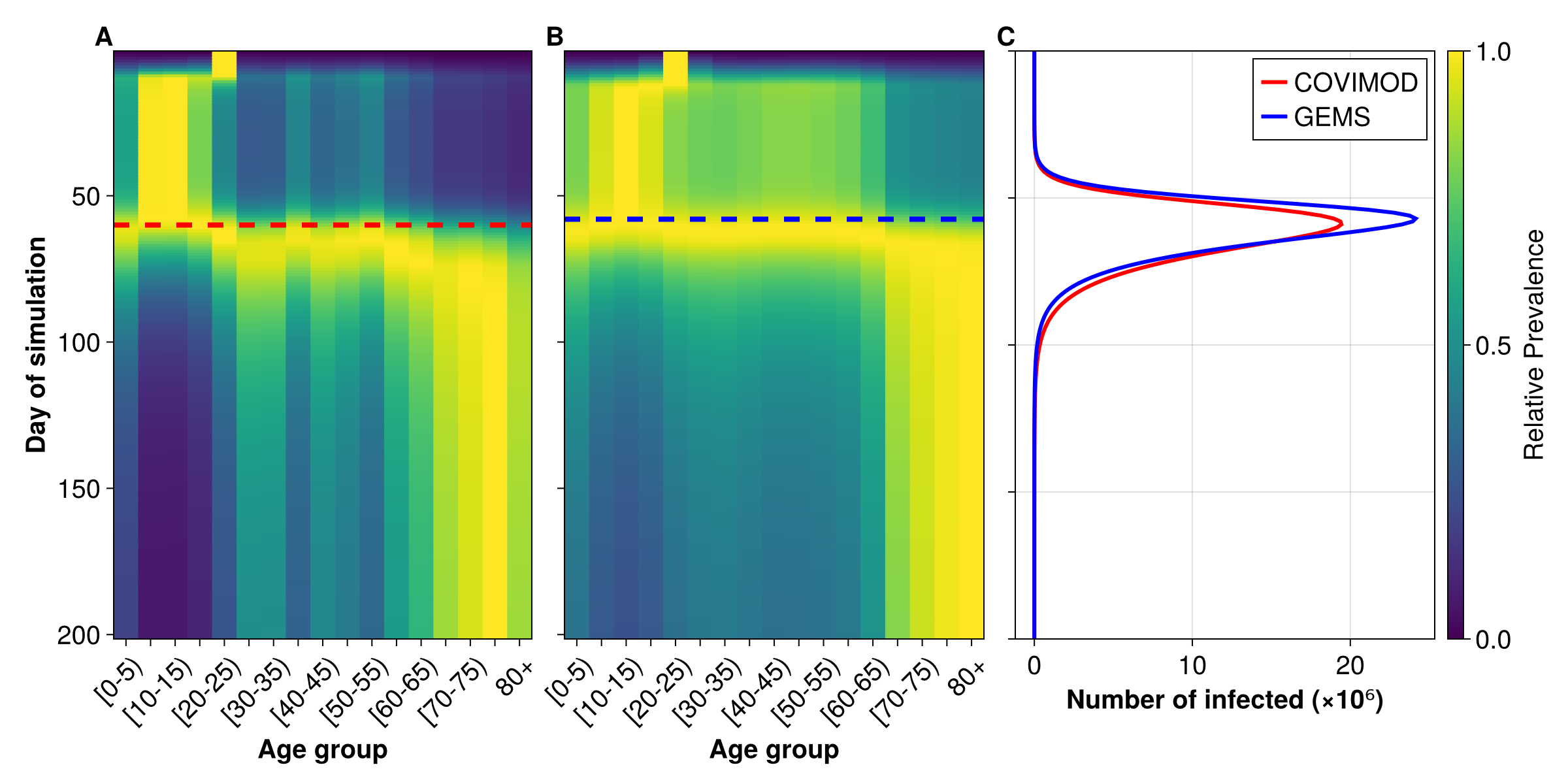


Fig E: SIR model results for group contacts derived from the populations age-distribution and an adapted beta value for the GEMS-based contact matrix. (A) and (B) display the relative prevalence in the age groups over the course of the simulation for the COVIMOD-based and simulated contact matrix, respectively. (C) displays the number of infected over the course of the simulation.

Also, the epidemic outcomes of the simulation, displayed in Table B, show a similar behaviour as for the results without group contacts. We obtain similar times of peak infections for both contact matrices while we obtain a higher attack rate for the GEMS-based simulation, highlighting the potential impact of using these different contact matrices for infectious disease models.

Table B: Differences in epidemic outcomes between the COVIMOD-based and simulated contact matrices including group contacts with population age distribution inference for the equal parametrisation and the modified beta for the simulated contact matrix. Negative values indicate higher outcome values of the simulated contact matrix.

| **Outcome Difference** | **Equal Parametrisation**  **(95% CI)** | **Modified Beta (95% CI)** |
| --- | --- | --- |
| Attack Rate | 0.08 (0.04; 0.11) | -0.08(-0.11; -0.05) |
| Peak Infections (10^6) | 7.4 (5.1; 9.3) | -4.7(-6.0; -2.7) |
| Peak Day | -51 (-58; -43) | -1.3(-6.0;9.0) |
| Final Susceptible (10^6) | -6.6 (-9.3; -3.7) | 6.8(4.1; 9.6) |

### Age-inference based on contact matrices

For the second inference method we assume that the age groups of group contacts of participants in a specific setting are similar to the age groups of individually reported contacts in that setting. Therefore, to sample the age groups we apply two steps. First, the contact matrices without group contacts, as displayed in the main text, are being calculated from the COVIMOD data. Second, for each person a participant has met in a group contact the contact matrix and the reported age group of the group contact is being used to sample the age group of this person. This is done by selecting the column of the setting-specific contact matrix corresponding to the participant age and restricting the possible age groups of the contact partner to the age group reported for the group contact. Then the age group of the contacted person is sampled from the relative contact frequency of the participant with each age group determined by the contact matrix. Note that if there are no contacts reported in any age groups that fall into the provided group contact age group, we assume equal likelihood for all possible age groups. For example, if 18-year old participant reported a group contact with 20 people aged 0 to 18 in a school setting, we use the contact matrix for schools without group contacts and determine the likelihood of contacting the age groups [0;5), [5,10) , [10,15) and [15,20), as defined by the relative number of contacts an individual in the age group [15,20) has with each of these age groups.

The resulting contact matrices in COVIMOD and GEMS are displayed in Fig F. Structurally the contact matrices are similar to the contact matrices in our main analysis. However, due to the sampling of the contact partners age the contact matrices show a higher number of contacts for all settings except the households. As before the main limitations of the represented contact structures in GEMS arise for the age-assortative mixing behaviour of young individuals, especially prominent for the school and other contacts.

The calibration of the setting-specific number of contacts leads to $\lambda_{H}=0.93$ for the household contacts, to $\lambda_{\text{office}}= {\lambda_{\mathrm{department}}=\lambda}_{\text{workplace}}{\approx0, \lambda}_{\text{workplacesite}}=2.10$, for the workplace, $\lambda_{\text{class}}=2.94, {\lambda_{\text{schoolyear}}=\lambda}_{\text{school}}=$ $\lambda_{\text{schoolcomplex}}\approx0$, for school contacts and $\lambda_{\text{O}}=1.78$ for other contacts. As before we report the global minima for the substructures other combinations are possible but lead to local minima. Similar to the analysis we performed without group contacts, we get only contributions for the school class while the less age-assortative substructures show no contributions. For the workplace, the minima are again all in a similar region and minor variations in contact behaviour between substructures leads to the contacts being best represented by the workplace site. These similarities to the main analysis are a result of the restrictive mixing patterns, imposed by using the contact matrix-based age inference of the group contacts. Using this procedure the existing structures in the contact matrices will be mostly kept since they provide the probabilities for sampling the age groups of the group contacts. However, when the age groups provided by the participants do not overlap with the main contributions in the contact matrices without group contacts larger changes in the contact matrices can be expected. For example, if a child would report a group contact with the 18-65 age group this would greatly impact the existing age-assortative structure since the group contacts would need to be sampled from the 18-65 age group. Since the matrices show no structural differences to the contact matrices in the main analysis there seems to be a large overlap between the age groups of individual contacts and the age groups of group contacts.


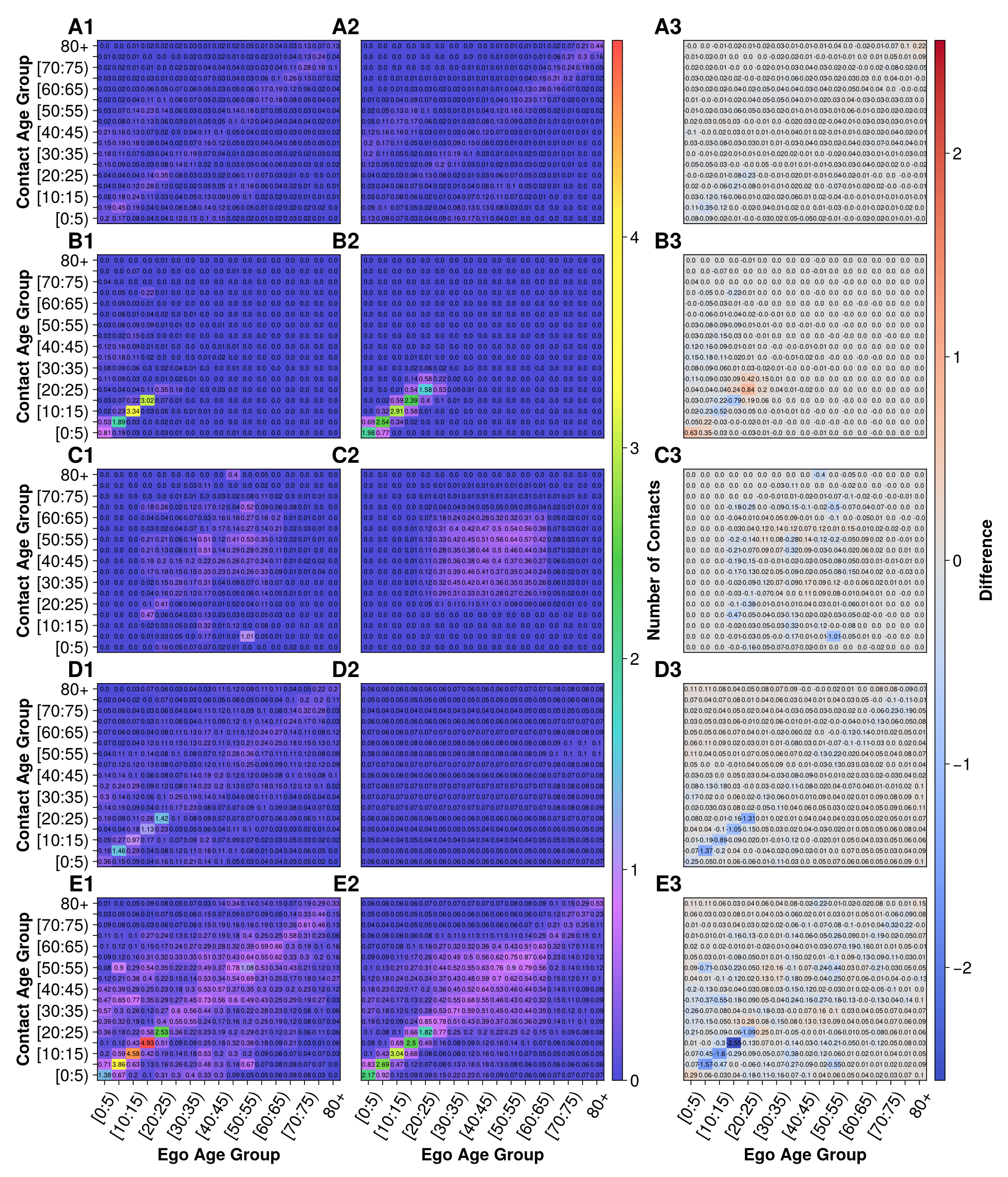


Fig F: Comparison of the age x age contact matrices for all contact settings using group contacts ages inferred from the contact matrices without group contacts. Each row corresponds to one contact setting, specifically they display (A) household, (B) school, (C) workplace, (D) other and (E) all contacts. The columns correspond to the (1) Contact matrix for the respective contacts derived from the COVIMOD contact survey, (2) Contact matrix for respective contacts based on the contact behaviour simulated in GEMS, and (3) difference between the COVIMOD and GEMS-based contact matrices for the respective contact setting.

The SSE and relative differences between the contact matrices using the contact matrix-based age inference are presented in Table C. Again, a similar trend as for the analysis without group contacts can be observed. However, similar to the age-distribution based inference these results also show increased SSE for all settings that include group contacts. Specifically, it leads to again to a 5-fold increase for school, 7.8-fold increase for workplace and 1.9-fold increase for other contacts. Compared to the age-distribution based inference all increases are larger which could be attributed to the stronger age-assortativity imposed by sampling the age based on the existing contact matrices. The relative differences remain constant, with only a small increase for school contacts.

Table C: Sum of squared errors (SSE) and mean relative difference between contact matrices in COVIMOD including group contacts inferred by the contact matrices without group contacts and the fitted GEMS calculated using Equation 1, with 95% confidence intervals determined by bootstrapping with respect to the COVIMOD participants.

| **Contact Setting** | **SSE (95% CI)** | **Relative Difference (95% CI)** |
| --- | --- | --- |
| Household | 0.7 (0.4 – 0.9) | 0.38 (0.36 – 0.40) |
| School | 3.5 (1.4 – 5.6) | 0.49 (0.46 – 0.51) |
| Workplace | 3.9 (0.9 – 6.8) | 0.47(0.46 – 0.49) |
| Other Contacts | 7.4 (3.5 – 11.4) | 0.29 (0.26 – 0.31) |
| All Contacts | 20.1 (10.2 – 30.0) | 0.22 (0.20 – 0.24) |

#### Epidemic Impact Analysis

The combined contact matrix yields a spectral radius of 6.45 and 4.46 for COVIMOD and GEMS, respectively. These radii lead to a reproduction ratio of  $\tilde{R}_{0}=1.45$, slightly smaller than for the inference using the age-distribution of the population and larger than without group contacts. Fig G and Fig H display the SIR model results with identical disease parameters and a modified beta value for the GEMS-based simulation, respectively. Again, a similar behaviour as before can be observed. The pathogen spreads mainly within the younger age groups prior to the peak of infections when the highest prevalence occurs in the older age group. Compared the COVIMOD-based simulation the GEMS-based simulation is more distributed over all age groups and shows a later peak in infections. Modifying beta for the GEMS-based simulation leads to a higher attack rate and peak infections as can be observed in Fig H and Table D. Further, Table D shows that the attack rate difference is substantially smaller than for the age group inference using the age-distribution and for the analysis without group contacts. This indicates that while the spreading process through the age groups remains different between the two simulation the resulting population-wide attack rate becomes more closely aligned.


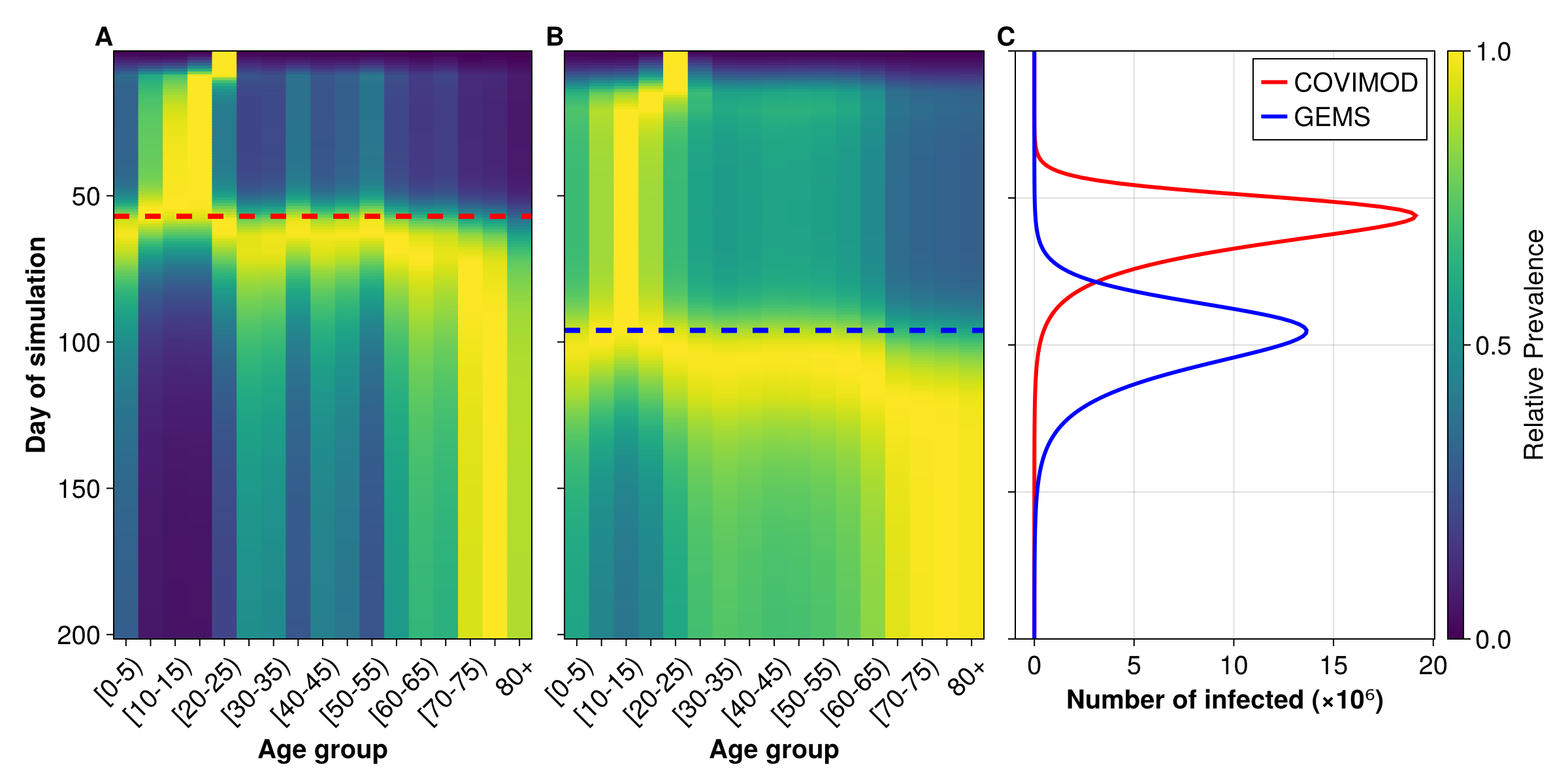


Fig G: SIR model results for group contacts age derived from the contact matrices without group contacts. (A) and (B) display the relative prevalence in the age groups over the course of the simulation for the COVIMOD-based and simulated contact matrix, respectively. (C) displays the number of infected over the course of the simulation.


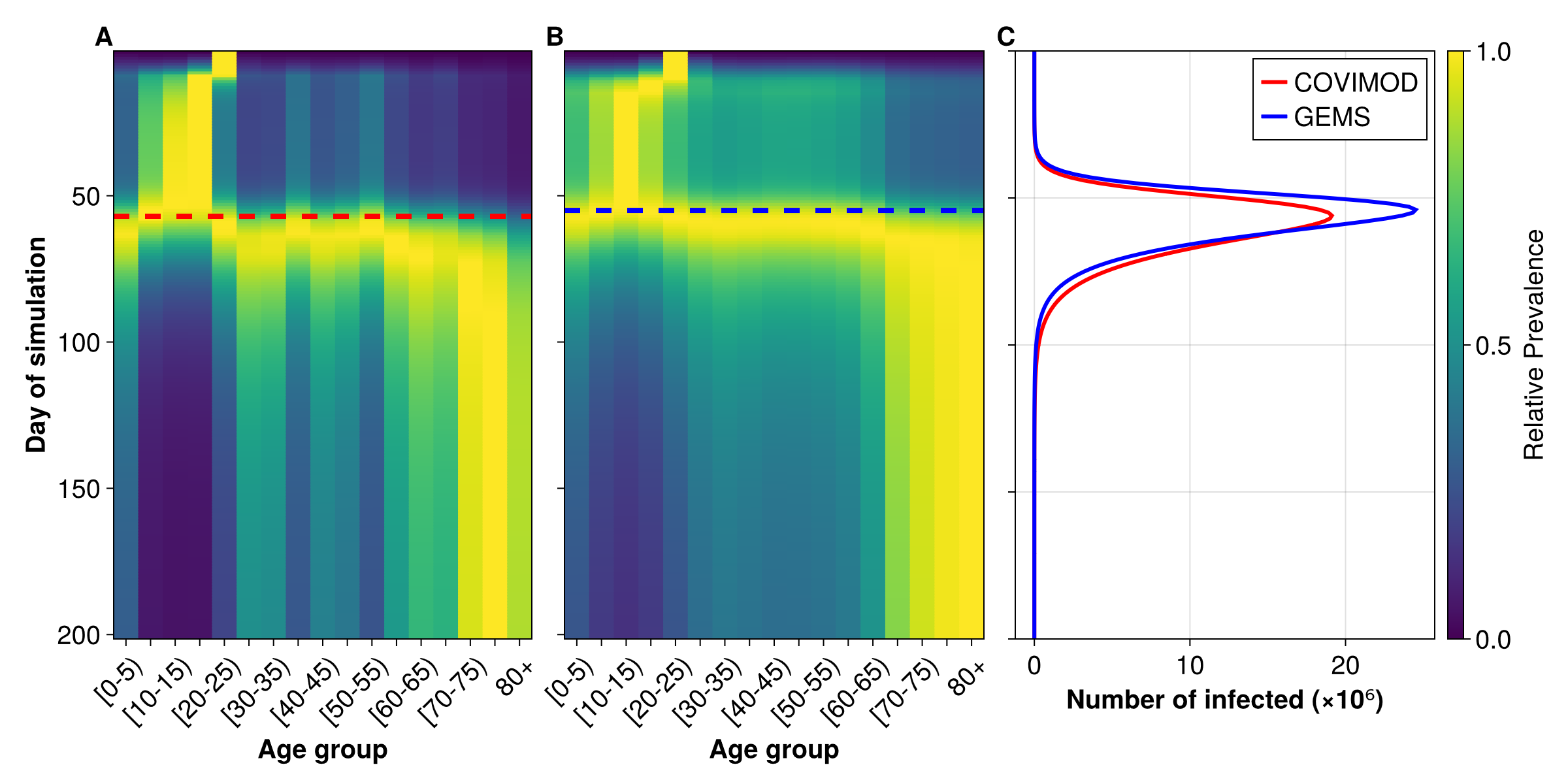


Fig H: SIR model results for group contacts age derived from the contact matrices without group contacts and an adapted beta value for the GEMS-based contact matrix. (A) and (B) display the relative prevalence in the age groups over the course of the simulation for the COVIMOD-based and simulated contact matrix, respectively. (C) displays the number of infected over the course of the simulation

Table D: Differences in epidemic outcomes between the COVIMOD-based and simulated contact matrices including group contacts with contact-matrix-based age inference for the equal parametrisation and the modified beta for the simulated contact matrix. Negative values indicate higher outcome values of the simulated contact matrix.

| **Outcome Difference** | **Equal Parametrisation**  **(95% CI)** | **Modified Beta (95% CI)** |
| --- | --- | --- |
| Attack Rate | 0.04 (0.01; 0.08) | -0.09 (-0.12; -0.05) |
| Peak Infections (10^6) | 5.4 (3.4; 7.8) | -5.4 (-7.5; -3.0) |
| Peak Day | -40 (-47; -32) | 1.33 (-5.53; 9.00) |
| Final Susceptible (10^6) | -3.7 (-7.0; -1.0) | -7.62 (-10.30; -4.38) |

## Clustering Results

Within Table E and Table F the results of the k-means clustering applied to the results of the numerical minimisation procedure using 10,000 random initial conditions for school and workplace contacts are shown.

Table E: Summary of clustering results from the numerical minimisation for the school contacts without group contacts. For each cluster (columns), median values of the scaling parameters across settings (rows) are shown, along with the respective cluster size and SSE value.

| Setting | C 1 | C 2 | C 3 | C 4 | C 5 | C 6 | C 7 | C 8 | C 9 | C 10 |
| --- | --- | --- | --- | --- | --- | --- | --- | --- | --- | --- |
| Size | 8357 | 313 | 285 | 262 | 369 | 35 | 32 | 23 | 303 | 21 |
| SSE | 2.43282 | 2.48196 | 2.43945 | 2.43726 | 2.45858 | 2.46655 | 2.59855 | 2.48621 | 2.43494 | 2.5405 |
| School Class | 0.00 | 0.00 | 0.95 | 0.62 | 0.00 | 0.47 | 0.22 | 0.83 | 0.30 | 0.00 |
| School Year | 1.10 | 1.14 | 0.14 | 0.46 | 1.15 | 0.67 | 0.21 | 0.26 | 0.79 | 1.45 |
| School | 0.00 | 0.73 | 0.00 | 0.00 | 0.37 | 0.45 | 0.00 | 0.66 | 0.00 | 0.15 |

Table F: Summary of clustering results from the numerical minimisation for the workplace contacts without group contacts. For each cluster (columns), median values of the scaling parameters across settings (rows) are shown, along with the respective cluster size and SSE value.

| Setting | C 1 | C 2 | C 3 | C 4 | C 5 | C 6 | C 7 | C 8 | C 9 | C 10 |
| --- | --- | --- | --- | --- | --- | --- | --- | --- | --- | --- |
| Size | 8143 | 281 | 290 | 272 | 40 | 4 | 310 | 63 | 272 | 325 |
| SSE | 1.2817 | 1.28252 | 1.28216 | 1.28187 | 1.28257 | 1.28268 | 1.28201 | 1.28218 | 1.28233 | 1.28175 |
| Office | 1.99 | 0.18 | 0.80 | 1.41 | 0.14 | 0.84 | 1.10 | 0.68 | 0.48 | 1.70 |
| Department | 0.00 | 1.81 | 1.18 | 0.57 | 0.42 | 0.20 | 0.88 | 0.62 | 1.49 | 0.28 |
| Workplace | 0.00 | 0.00 | 0.00 | 0.00 | 0.00 | 0.82 | 0.00 | 0.00 | 0.00 | 0.00 |
| Workplace Site | 0.00 | 0.00 | 0.00 | 0.00 | 1.31 | 0.29 | 0.00 | 0.74 | 0.00 | 0.00 |

## Epidemic Impact

To assess the spread of pathogen through the simulated age groups we ran the age-structured SIR model for different initially infected age-groups. Fig I displays the simulation results for different initially infected age groups for both the COVIMOD-based contact matrix (1) and the GEMS-based contact matrix (2). (A) corresponds to one person in the [0,5) age group to be initially infected, (B) to one person in the [5,10) age group and so on. While the initially infected group varies the overall behaviour is similar. The pathogen spreads within the initially infected age groups due and quickly moves towards the younger age groups within which it spreads until the peak. After the peak it is mainly prevalent in the older age groups. For the GEMS-based contact matrix the prevalence is more evenly spread over all age groups, while the general behaviour is similar to the COVIMOD-based contact matrix.


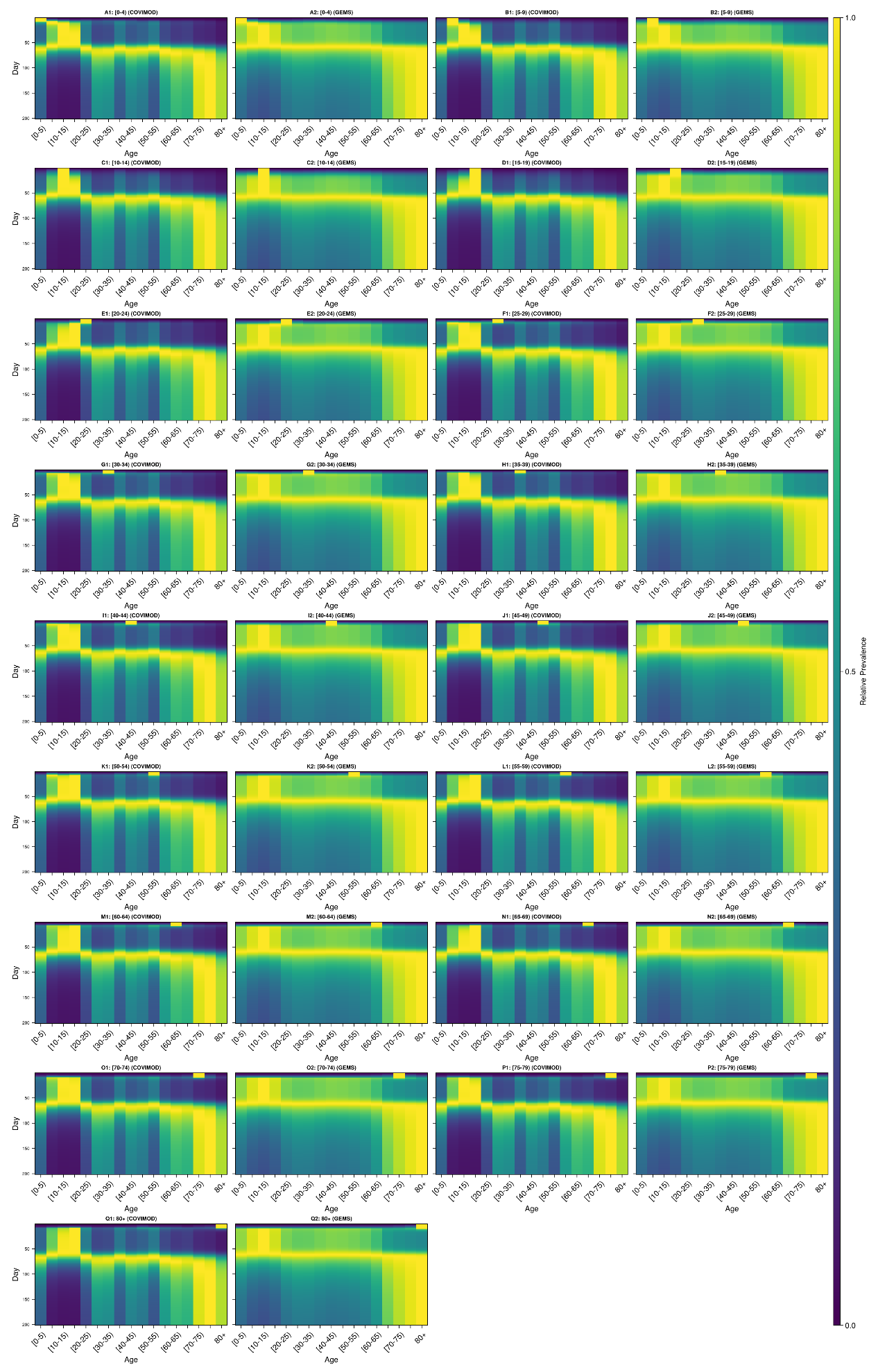


Fig I: Relative prevalence of the pathogen during a set of simulations of an age-structured SIR model with different initially infected groups. Letters indicate the different initially infected groups while 1 indicates the COVIMOD based contact matrix and 2 the GEMS based contact matrix. During each simulation one individual of the respective group was initially infected.
