# Supplementary material for: A comparison of contact patterns derived from the population structure in agent-based models and empirical contact survey data
Source: PLoS Comput Biol. 2026 Jun 18;22(6):e1013533. doi: 10.1371/journal.pcbi.1013533 (PMC13309034; doi:10.1371/journal.pcbi.1013533)
Supplement: Attachment — Submitted filename: revision_letter.pdf [file pcbi.1013533.s002.pdf]

Reviewer #1:

Reviewer #1:

This manuscript presents a thoughtful and well-executed comparison between contact structures simulated by an agent-based model (GEMS) and those observed in the empirical COVIMOD contact survey. By evaluating how the random mixing assumption performs across different settings—households, schools, workplaces, and other environments—the study contributes to improving the realism and interpretability of epidemic models. The work addresses a timely and relevant topic for computational epidemiology. However, several methodological and interpretative aspects could be clarified or extended to strengthen the conclusions. Addressing these points would make the manuscript a more robust and impactful contribution to the literature.

I list my comments here below, listed in order of importance and divided between "major" and "minor".

Major Comments

1) The authors rightfully acknowledge that their method does not incorporate age-specific differences in contact activity and that this is a big limitations of their work. Since such heterogeneity is a fundamental feature of human contact patterns, its omission substantially limits the model's realism. The authors could explore introducing an age-dependent calibration factor for contact frequency. This extension (although more parameterized) should be computationally manageable and could greatly improve model fidelity. If implementing this is not feasible, it would be helpful to explain the technical constraints preventing it.

Response:

We thank the reviewers for highlighting this issue. We agree that using an identical number of contacts for all members of a setting is a limitation of the implemented random mixing assumption. There are two main reasons why we consider this age-independent contact sampling method. First, we want to assess this contact sampling method without age-dependent contact numbers since it is commonly employed in large-scale ABMs. Therefore, introducing a more complex sampling method might improve the representation of real-world contact behaviour but will make our ABM (GEMS) less representative of other large-scale ABMs. Second, we want GEMS to continue to be a computationally feasible model for large populations. Although GEMS allows for age-dependent contact sampling this slows down the simulation, especially for large populations and settings. This is a consequence of contact probabilities depending on the attributes of both contact partners. Achieving the desired contact distribution requires more than sampling the desired number of contacts for an individual (ego) randomly from the available population (alters), instead the contact probabilities of the alters must also be considered. Therefore, for a feasible simulation of large populations it is still the most reasonable approach to use the random mixing assumption. However, we acknowledge that for simulating smaller populations the more suitable choice are complex contact sampling methods that more closely reflect the real-world contact structure, such as the age-dependent contact sampling suggested by the reviewers.

To reflect these considerations in our manuscript we extended the conclusion to include the computational considerations that went into the choice of contact sampling method and highlighted the usage of such a sampling method by other large-scale ABMs. Specifically, we mention the increased computational complexity of approaches where the likelihood of a contact depends on the properties of both contact partners for large-scale ABMs such as the one used within our analysis these approaches are often avoided to maintain computational tractability. This includes the point raised on age-dependent contact numbers. The changed text passages are:

"The representation of heterogeneous contact behaviour requires methods that include household size dependent number of contacts and contact probabilities dependent on attributes of the potential contacts, such as their age. Similar attribute-based approaches could address the limitations in representing the other contacts. However, such attribute-based approaches would drastically increase the model's computational complexity, as the probability of sampling an individual as a contact would depend on the properties of both contact partners. One simplified solution could be the implementation of two independent subsettings of which one captures age assortitiveness while the other does not." [line 631 - 638].

Reviewer #1:

2) The decision to compare empirical and simulated contact matrices is appropriate and informative. However, the interpretation remains largely structural, without exploring how these differences may affect epidemic potential. To strengthen the manuscript's epidemiological relevance, I encourage the authors to use a next-generation matrix approach to derive and compare the basic reproduction numbers (R_0) from the COVIMOD and GEMS matrices. This addition would contextualize the observed discrepancies in a more interpretable and policy-relevant way, since identical matrix differences can have markedly different effects on R_0 depending on the age pairs involved. It could be that the discrepancies observed between COVIMOD and GERMS matrices do not have a striking epidemic impact.

Response:

We thank the reviewer for this valuable suggestion. We agree that linking the identified matrix differences to epidemic outcomes is essential, and we have substantially revised the manuscript to address this point.

We quantify the epidemiological consequences of using the COVIMOD versus the ABM-derived (GEMS) contact matrices using two methods:

1. Next-generation matrix analysis: We compute and compare the basic reproduction numbers implied by each matrix for an age-structured SIR-model and report a disease-independent reproduction number ratio. This ratio is substantially above one, indicating that the two matrices lead to substantially different transmission dynamics.
2. Age-structured SIR simulations: In line with the reviewer's recommendation to use a compartmental model (and not the ABM) as the model, we implemented a simple age-structured SIR model in which the contact matrices determine age-group mixing. We examine key outcomes, including final attack rate, peak incidence, and age-specific dynamics. The matrices yield different epidemic outcomes and visibly different spread patterns through the age groups. Notably, after adjusting transmission so that both simulations have the same

reproduction number, the ABM-based matrix produces a higher total number of infections.

Together, these analyses suggest that the observed structural differences between the survey- and ABM-based matrices can translate into substantial differences in disease dynamics within our employed SIR-model. Our results also suggest that relying on random-mixing assumptions and calibrating solely to match empirical reproduction numbers could overestimate transmission potential. However, we note that by comparing the simulated and COVIMOD-based contact matrices using the SIR-model and the next generation matrix, we assumed that COVIMOD captured all contacts relevant for disease transmission and that those contacts had an equal probability of transmission. Because both assumptions are only approximations, we cannot be certain that using ABMs with random mixing in settings actually leads to substantial differences in infectious disease dynamics from real-world dynamics. We believe these additions strengthen our manuscript, specifically the epidemiological interpretation of our findings.

Due to the extensive changes in various chapters, we will only display the text of the changes in the conclusion section here. The remaining changed text passages can be found in the methods section under “Epidemic Impact Analysis” (line 306-337), in the results section titled “Epidemic Impact Analysis Based on a Compartmental Model” (line 522 - 574), and in the conclusion section (line 601-621, line 698-711):

“The comparison of the resulting contact matrices using an age-structured SIR-model indicates that the differences in the contact matrices could translate into substantial differences in the epidemic outcomes. However, it has to be kept in mind that this comparison is conducted in a compartmental model which cannot represent relevant dimensions of an ABM. The results translate therefore not necessarily to what can be expected in the ABM itself. Using the next-generation matrix approach resulted in a reproduction number ratio substantially larger than one, corresponding to a faster disease spread when using the survey-based compared to the simulated contact matrix in the SIR-model. We also observed differences of the disease dynamics simulated in the SIR-model, including differences in outcomes such as the final attack rate and, peak infection numbers. In addition to these aggregate epidemiological indicators, the two contact matrices produced broadly similar but still noticeably different distinct spreading dynamics between age groups. These differences became more apparent when both simulations were adjusted to equal reproduction numbers. We obtain a similar impact on the infectious disease simulation when we include group contacts.

Together these findings suggest that applying a population structure-triggered contact behaviour in ABMs may lead to unintended consequences when modelling infectious diseases. However, our comparison is based on a simple SIR model, which does not take into account factors such as contact clustering or repeated interactions which influence transmission dynamics both in the real-world and in ABMs. Therefore, it is unclear to what extent the identified differences also correspond to differences between real-world dynamics and those simulated in ABMs.” [line 601-621],

“As described above, the epidemic impact analysis performed has to be interpreted with caution. While we identify mismatches of the transmission dynamics when parametrising an SIR model with the COVIMOD-based vs the simulated contact matrix, these mismatches do not necessarily translate to mismatches between real-world infections and infections simulated in the “full” ABM. Within our analysis we use the SIR model as an approximation of both the real-world and the ABM. This approach implicitly assumes that contact matrices alone suffice to represent infections and neglects features such as the clustering of contacts which we know to influence real-world and simulated disease dynamics. Furthermore, we assume that COVIMOD

with its previously discussed limitations accurately captures all contacts relevant for disease transmission. We know that these assumptions are only approximations and do not hold exactly. Consequently, our results do not necessarily translate to mismatches between the transmission dynamics in the real-world and "full" ABMs. Rather, our results suggest that mismatches might exist and care should be taken to accurately assess their presence and impact." [line 698-711].

Reviewer #1:

3) The imputation of missing ages for group contacts using the national population age distribution is a practical choice, but location-specific age structures might provide a more accurate representation. I suggest reporting the proportion of group versus individual contacts (to put this problem in perspective) and exploring how alternative imputation strategies (for instance, setting-specific age structures) influence the results.

Response:

We thank the reviewer for this comment. We understand that our manuscript requires some clarification on how group contacts were handled. Our initial analysis in fact excluded group contacts, since these were only reported in coarse age brackets (0–18, 18–65, 65+), while the individual contact partners were reported in 5-year age bands. To assess how this decision affects our results we extend our analysis by using two different methods for imputing the age of group contacts and conduct analyses identical to the one for the COVIMOD data without group contacts.

Specifically, we apply two imputation strategies: (i) we impute exact ages from the provided age groups and the population age distribution, and (ii) a two-step setting-specific approach where first we calculate the setting-specific COVIMOD contact matrices derived from individual contacts without group contacts. Second, we use these matrices to impute 5-year age bands each reported group contact using the participant age, the reported group contact age group and the contact matrix entries for the corresponding age groups.

When we compared the COVIMOD contact matrices with group contacts and the GEMS contact matrices, we observed a behaviour similar to the COVIMOD contact survey without group contacts. Again, the main differences for household and school settings lie in the different social activities of different age-group and the largest SSE between the COVIMOD and GEMS contact matrices arises for the other contacts. However, especially for the age imputation using the population age-distribution the SSE for schools and workplaces increased by a larger factor than the SSE for the other contact. This may be due to an overall increase in the number of contacts, as well as age sampling, which is similar to sampling for other contacts. In both cases, contact partners are randomly selected, thereby following the age distribution of the population.

We have clarified the description of how we handle group contacts in the methods section of our manuscript. We report our analyses using the two different age-imputation methods in our Appendix titled "Group Contacts". There, we also report the setting-specific share of group contacts. For brevity we refer to the appendix for these changes. The changes in the COVIMOD description of the methods section are:

"In the COVIMOD survey, participants could also report group contacts in settings other than the household. For these group contacts, participants had to report the number of individuals they had met and their collective age group. These age groups were defined as under 18, 18–64, and 65 or over. However, due to the coarse resolution of these age groups, obtaining accurate mixing

matrices is not trivial. Since participants only reported these coarse collective age groups, it is not possible to construct the contact matrices with five-year age bands from group contacts and individual contacts combined directly. While it would be possible to map the individual contacts reported in five-year age bands to the age groups of the group contacts, doing the opposite requires the imputation of the age groups of the group contact partners. There are different approaches to impute the age groups of the group contacts; however, they need to be based on assumptions to generate the more granular age groups [24,25]. As the COVIMOD study without group contacts already includes a large sample of contacts and we are more interested in the structure of the represented contacts than the actual number of contacts we consider only individually reported contacts and exclude group contacts in the main analyses. However, we present analogous analyses using two different methods for imputing the age of group contacts in the appendix. This allows us to assess the impact of our decision to exclude group contacts from the analysis." [line 144-160].

Reviewer #1:

4) The assumption that all sub-locations (e.g., school classes, years) are equally likely contact environments simplifies reality and may overlook important structural heterogeneity. For example, contact intensity is typically highest within classes and lower between them (so the lambda for classes should be larger than the one for the year, contrary to the current results). Furthermore, the calibration procedure leads to hierarchical λ values across sub-locations, although their age structures appear similar. It would be helpful for the authors to elaborate on why this hierarchy arises, since I would expect all sub-locations lambdas to be equal, if the underlying structure of mixing at random is the same.

Response:

We thank the reviewers for raising this point. Since we employ a numerical minimisation procedure to identify the best-fitting contact numbers for each substructure the respective lambda values are derived solely based on the contact matrices of COVIMOD and the age-constellation of the substructures in GEMS. As schoolyears and classes have almost identical age-constellation, there is no feature that would cause the numerical minimisation procedure to favour the class substructure. Instead, minor differences in the age-constellation seem to result in a better representation of the COVIMOD contact structure when using the schoolyear substructure.

To incorporate a more objective assessment of the obtained values from the minimisation procedure and identify the reasons why they arise, we extended our analysis by performing multiple minimisations with random initial conditions and incorporating a k-means clustering of the solutions. Because the numerical minimisation can converge to local minima, clustering the resulting minima allowed us to systematically identify distinct groups of solutions, each representing a different local minimum with its own combination of rescaling parameters and associated SSE. This analysis revealed that several parameter combinations can fit the data reasonably well, but they differ in how they distribute the contacts across sub-locations. Examples include the combination of contacts in the schoolyear and class substructures, which led to similar but slightly higher SSEs than for only using the schoolyear. Importantly, the contact-hierarchy (the lambda values for the substructures) reported in the original manuscript corresponds to the global minimum: it is the solution most frequently recovered across minimisation runs and yields the lowest SSE. The alternative hierarchies arise from local minima but are less common and provide a worse fit.

We have revised our manuscript to report this additional analysis. Specifically, we report the clustering method in the methods section:

"To assess the numerical stability of the solutions we run the minimisation procedure 10,000 times with random initial conditions followed by a k-means clustering implemented in the Clustering.jl package in Julia [39]. This allows us to identify the different local minima and estimate the global minimum. For the further analysis, we use the global minimum to define the contact numbers for the respective substructures in the ABM." [line 287-291],

and the different possible combination of lambda-values and their respective frequency during the optimisation process in the results section for school contacts:

"The contact matrices for all substructures of the school using the uncalibrated random contact sampling are displayed in Figure 8 in the Appendix. Here, the school classes and school years represent highly age-assortative mixing while schools and school complexes also capture contacts between age groups. Calibration of the average number of contacts for the substructures yields: $\lambda_{schoolyear} = 1.26$ *and* $\lambda_{class} = \lambda_{school} = \lambda_{schoolcomplex} \approx 0$*. As expected, the age structure of school years alone most closely replicates the contact structure observed in COVIMOD. Note that these average numbers of contacts correspond to the most common solution of the numerical minimisation. Using the k-means cluster analysis of the minimisation results with random initial conditions we identified this kind of solution as the global minimum being obtained 88 % of times. The remaining minima correspond to local minima. Most common were combinations of school classes and school years, i.e., where* $\lambda_{class} \neq 0$ *and* $\lambda_{schoolyear} + \lambda_{class} \approx 1.26$*. Less than 0.3% of solutions also show contributions of the school setting. Further information on the identified clusters is displayed in Table 7 in the appendix. Looking at the values of Equation 2 for these solutions shows that* $\lambda_{schoolyear} = 1.26$ *is the global minimum, indicating that the small differences in the contact structures of the substructures leads to the best fit when only the schoolyear is included. In the following we use the contact numbers corresponding to this global minimum. The calibrated ABM school contact matrix is displayed in Figure 2 (B)."* [line 385-402],

and in the workplace section:

"The contact matrices for the four workplace substructures using the uncalibrated random contact sampling are presented in Figure 9 in the Appendix. As all substructures have a similar age structure, i.e., the age-constellation of workers in an office is on average identical to the age-constellation of entire companies, all substructures also exhibit a similar contact structure. The calibration of the contact numbers of the substructures with the COVIMOD data results in $\lambda_{workplace} = 0.29$*,* $\lambda_{department} = 0.81$ *and* $\lambda_{office} = \lambda_{workplacesite} \approx 0$*. As for the school contacts, we used k-means clustering to identify that this solution corresponds to the global minimum of the SSE. It was obtained in 91% of the runs. Most remaining solutions correspond to other combinations of workplace and department with* $\lambda_{workplace} + \lambda_{department} \approx 1.1$*. In 3% of cases there are contributions from the office. Note, that the SSE differences between clusters displayed in Table 8 of the appendix are much smaller for the workplace substructures than for the school substructures due to the similarity of the age composition and contact structures across substructures. In the following we will again use this global minimum for our analysis."* [line 428-440].

We present the raw clustering results as Tables 7 and 8 in the appendix.

Reviewer #1:

Minor Comments

1) Line 190: The period should be replaced by a comma; otherwise, the clause beginning with "using" lacks a clear subject.

Response:

The sentence was rephrased accordingly. See:

"While we use GEMS for our comparison, other ABMs follow the same methodology: associating population members to specific locations, in which random contacts are modelled [6,16,36,17]." [line 253-254].

Reviewer #1:

2) The manuscript reports small deviations for home, school, and workplace settings. Could this partly reflect the smaller number of contacts in these settings compared to "other" contacts? Reporting relative errors, in addition to absolute differences, could clarify this point and might provide a better sense of the epidemiological significance of these discrepancies (see major comment 2).

***Response*:**

We thank the reviewer for this valuable suggestion. In response, we have expanded our analysis to include mean relative differences between simulated and empirical contact numbers. As the reviewer suggested, the 'other' contacts were 'relatively' well reproduced, as is now clear when examining relative differences. However, we would like to emphasise that, while a relative comparison can provide useful insight into proportional deviations, the epidemiological relevance is primarily driven by absolute contact numbers rather than relative differences alone.

We address the reviewers' comment in the manuscript by reporting the relative differences as a second quantity besides the SSE to compare the contact matrices of COVIMOD and GEMS. Specifically, we added the formula for the relative difference to the Methods section:

"For quantitative comparison, the minimal SSE given by Equation 1 and 2, and mean relative difference given by:

$$D(M_C, M_S) = \frac{1}{N_{age}^2} \sum_{i,j} \frac{|m_{ij}^C - m_{ij}^S|}{m_{ij}^C + m_{ij}^S}, (Equation\ 3)$$

are used as measures of the difference between the two contact matrices. Here N_{age} corresponds to the number of age groups which in our case is 17." [line 296-300],

reported the relative differences in the Results:

"Table 1 presents the SSE and mean relative difference between the ABM population-generated and COVIMOD contact matrices for each contact setting calculated using Equation 1 and Equation 3, along with the 95% confidence intervals for the obtained through bootstrapping with respect to the COVIMOD participants. Household, school, and workplace contacts all show similar SSE values: 0.7 (95% CI: 0.4 to 0.9), 0.7 (0.3 to 1.1) and 0.5 (0.2 to 0.7), respectively. In contrast, the "other contacts" show a considerably higher SSE of 3.8 (1.2 to 6.5). Lastly, the deviations for all contacts lead to an SSE of 7.6 (95% CI: 2.3 to 13.0). The mean relative difference shows a trend opposite to the SSE, with the other contact and all combined contacts displaying a smaller mean relative difference with 0.29 (0.27 – 0.32) and 0.20 (0.18 – 0.22), respectively. Household, school, and workplace contacts all show differences around 0.4, which is higher but still in the same region as the other and all combined contacts. This reversed trend stems from larger numbers of average daily contacts between the age groups in the other and all combined contacts. Note, however, that for the spread of a pathogen relative differences are of lesser

importance than absolute differences. At the same time, different transmission probabilities per setting, particularly with lower probabilities in other places would diminish the difference.

Table 1: Sum of squared errors (SSE) and mean relative difference between contact matrices in COVIMOD and the fitted GEMS calculated using Equation 1, with 95% confidence intervals determined by bootstrapping with respect to the COVIMOD participants.

Contact Setting	***SSE (95% CI)***	***Relative Difference (95% CI)***
Household	*0.7 (0.4 - 0.9)*	*0.36 (0.34 – 0.38)*
School	*0.7 (0.3 – 1.1)*	*0.44 (0.42 – 0.46)*
Workplace	*0.5 (0.2 – 0.7)*	*0.45 (0.43 – 0.47)*
Other Contacts	*3.8 (1.2 – 6.5)*	*0.29 (0.27 – 0.32)*
All Contacts	*7.6 (2.3 – 13.0)*	*0.20 (0.18 – 0.22)*

“ [line 502-521],

and incorporated a short discussion of their implications into the Conclusion:

“This is a result of the chosen implementation of the ABM, which models “other contacts” as random contacts in the municipality. This approach fails to account for the heterogeneous contact behaviour observed in COVIMOD. While the trend is less pronounced when considering group contacts, it is still prevalent. Looking at the relative differences between the contact matrices derived from COVIMOD and the population structure of the ABM reveals an opposing trend with all differences being of the same order of magnitude but with “other contacts” showing the smallest differences. This opposite trend is a result of the high number of daily contacts in the other category. It highlights the importance of correctly representing other contacts since even as they are incorporated relatively well the SSE still shows the largest deviations for these other contacts. At the same time, the contacts in other settings could play a smaller role epidemiologically due to lower transmission probabilities during a contact.” [line 590-600].

Reviewer #1:

In conclusion, this is an important and promising contribution to the ongoing discussion on how best to represent contact structures in large-scale agent-based models. By expanding the paper to quantify epidemiological consequences, incorporating age-specific contact activity, and clarifying several modelling assumptions, the paper would achieve a stronger impact and broader relevance for the field.

***Response*:**

We thank the reviewer very much for the time taken to review our manuscript and for the very constructive and encouraging comments.

Reviewer #2:

Reviewer #2:

Thanks for the opportunity to review this manuscript.

In their work, the authors compare the contact matrices obtained from a large contact study in Germany between 2022 and 2023, with the contact matrices obtained from a synthetic population of Germany, used in a publicly available agent-based model.The work is interesting, and it addresses an important research question. There is much research work that compares agent-based models and survey contact matrices, however, most of these studies date back to the POLYMOD study (used as a

benchmark). Few studies have tackled this problem after COVID-19 and I see this paper as a nice contribution.I have one major remark that I consider important to address and a minor comment. In general, my main concern is that the paper does not engage sufficiently with the consequences of its findings on the epidemiology of respiratory infections, and disease dynamics.

Major issues:

- The study exposes in detail the differences between contact matrices from survey and ABM but a fundamental question remains unanswered: what is the impact of such (relatively small differences) on simulated epidemics? This is a key question that naturally arises from the results. More specifically, I suggest comparing the results of a simulated SEIR stochastic model that uses either matrix, for different assumptions on disease transmissibility. Does the final attack rate varies significantly? Please, note that I am not suggesting using the ABM as the epidemic tool. Instead, simulate an age-structured compartmental model with different matrices.

- Related to the above point, the contact matrix can be directly linked to the basic reproductive number, R_0, in a model through its spectral radius. Are the spectral radiuses of the matrices very different? What is the difference in R_0 that we expect to observe given the different matrices?

Response to both comments above:

We thank the reviewer for this valuable suggestion. We agree that linking the identified matrix differences to epidemic outcomes is essential, and we have substantially revised the manuscript to address this point.

In the revised version, we added a new section “Epidemic Impact Analysis” (line 306-337) to the methods section, a section titled “Epidemic Impact Analysis Based on a Compartmental Model” (line 522 - 574) to the results section, and multiple paragraphs to the conclusion (line 601-621, line 698-711). Since these changes include entirely new sections and we want to keep this document at a readable length, we do not display the changes here but rather refer to the changed document lines. We quantify the epidemiological consequences of using the COVIMOD versus the ABM-derived (GEMS) contact matrices using the two methods suggested by the reviewer:

a. Next-generation matrix analysis: We compute and compare the basic reproduction numbers implied by each matrix within an age-structured SIR-model and report a disease-independent reproduction number ratio. This ratio is substantially above one, indicating that the two matrices lead to substantially different transmission dynamics.
b. Age-structured SIR simulations: In line with the reviewer’s recommendation to use a compartmental model (and not the ABM) as the model, we implemented a simple age-structured SIR model in which the contact matrices determine age-group mixing. We examine key outcomes, including final attack rate, peak incidence, and age-specific dynamics. The matrices yield different epidemic outcomes and visibly different spread patterns through the age groups. Notably, after adjusting transmission so that both simulations have the same reproduction number, the ABM-based matrix produces a higher total number of infections.

Together, these analyses demonstrate that the observed structural differences between the survey- and ABM-based matrices can translate into substantial differences in disease dynamics within our utilised SIR-model. Our results also suggest that relying on random-mixing assumptions and calibrating solely to match empirical reproduction numbers could overestimate transmission potential. We believe these additions strengthen our manuscript, specifically the epidemiological interpretation of our findings.

Reviewer #2:

Minor issues:

- On what data the synthetic population of GEMS is based? Is census data from the latest national census? What year does it represent? I might have missed this information from the paper. Results could be partially explained by the recency of the ABM population data.

Response:

While our original manuscript included a short description of the synthetic population used, called Gesyland, we greatly extended this description to include more details on the data sources and methods used. Furthermore, we reference a newly published article of the technical description of the synthetic population construction methodology. The adapted description can be found in the methods section:

"The structure of contacts in GEMS is a result of the association of individuals to settings. The associations with settings are determined during the generation of the Gesyland population [29,30], a synthetic population that represents various characteristics of the German population. The locations and types of settings, such as home locations, schools or workplaces, are obtained from OpenStreetMap [31]. The home locations are filled with households, where the household size is obtained from the household size distribution based on the German Census 2011 [32]. This Census data is available at a 100m square grid, allowing for regional variations in household size distribution. Individuals in the households are created probabilistically based on demographic tables within household size classes. Further variables like education, occupation and health related variables obtained from the German microcensus 2019 [33] and the German health survey GEDA (2019/2020-EHIS) [34], and added via a predictive random forest procedure. The assignment of individuals to schools and workplaces is based on individual characteristics and the interregional commuting structure derived from commuting tables [35]. Substructures in schools are created based on the structure of the school system. Schools consist of school years which consist of classes. Additionally multiple school may share the same common facilities and are structured in a school complex. For workplaces substructures are determined by randomly creating smaller groups of the workers in a workplace and thereby create departments and offices. Workplaces sharing the same location are grouped in a workplace site. While producing the individual properties and affiliations, the known constraints on higher levels are kept. These constraints included among others the total number of employed people in a region and the number of people having a certain educational level. Hence, the hierarchical population structure is consistent to known totals obtained from the German Census 2011 [32] regarding the households' structure, and consistent to estimated totals for all other variables according to the German microcensus 2019 [33] and German health survey GEDA (2019/2020-EHIS) [34]. A dedicated description of the Gesyland population and the method used for its creation elsewhere [30]." [line 227-252].

Reviewer #3:

Reviewer #3:

The manuscript by Suer et al addresses on what extent the contact behavior modeled by agent-based model well reproduce real data. Agent based models build a synthetic population which capture the collocation of individuals in same settings – households, workplaces, schools, and other settings. The manuscript addresses the comparison between modelled and empirical contacts, where the latters are obtained from national surveys. It shows that modelled contacts in setting characterized by a well-defined age composition reproduce the basic properties of real patterns. Contacts outside households, schools, and workplaces, present the largest deviation with empirical data.

The manuscript is not clearly written. The importance of the work relative to the existing literature is not discussed. Finally, the analysis is poor. Therefore, I cannot recommend the publication of the work in the present form. Substantial revisions are necessary before the work can be considered suitable for publication.

More specifically:

The manuscript is not clearly written. Sentences are unclear in many parts. The analysis done is not clearly presented.

Response:

We have thoroughly revised the manuscript to improve clarity and readability throughout. Specifically, we have: Restructured unclear sentences and simplified complex phrasing where possible, ensured consistent terminology and logical flow between sections, incorporated suggestions from a native English speaker to improve grammar and style and paid particular attention to the methods and results sections to ensure that our approach and findings are presented as clearly as possible.

We hope these revisions have improved the manuscript's clarity and hope the reviewer finds the revised version more accessible.

Reviewer #3:

Not enough background is provided on the agent-based model used in the study; therefore, it is not clear how the matrices are reconstructed and calibrated from the data. For instance, I could not understand the procedure for contact sampling in GEMS described in line 170-179 (why contacts are drawn with replacement?),

Response:

We thank the reviewer for highlighting the need for greater clarity on the methodology of the agent-based model. With regard to the question on contact sampling: We sample contacts with replacement to reduce the computational cost of contact sampling. Sampling without replacement would increase the computational cost, particularly in large settings such as municipalities or workplaces, since the cost increases with the number of individuals present rather than the number of contacts drawn.

In the revised manuscript, we have substantially expanded the background on the ABM, provided the rationale for sampling with replacement and clarified how contact matrices are reconstructed, with the following key changes:

- Calibration procedure: We renamed the Methods section from “Comparison” to “Calibration and Comparison” (line 260-305) and emphasized that the contact numbers obtained through the minimization step are the contact parameters used in our simulations. We now explicitly describe how these parameters are derived and subsequently applied within the model:

  “As the initial parametrisation of the random contact sampling in the ABM, the average number of contacts per day is set to one, i.e., $\lambda_T = 1$ for all setting types T in the simulation. Based on the simulated contact matrix M_S^T and the contact matrix derived from COVIMOD M_C^T the average number of contacts λ_T is calibrated as follows. Note that to simplify the notation, from now on we drop the setting type index T, while still referring to setting type specific variables. Since the entries in the contact matrix scale linearly with the defined average number of contacts λ, scaling the average number of contacts by α, such that $\lambda' = \alpha\lambda$ results in a linearly scaled contact matrix $M_S' = \alpha M_S$. As we want to align the contact structure in the ABM with that observed in the contact survey, we choose to minimize the difference between the two contact matrices to obtain the calibrated contact numbers λ' for the ABM. To describe the difference between the two contact matrices, we use the sum of squared errors (SSE), placing more emphasis on large deviations than small ones. The SSE for the survey-based contact matrix M_C and the linearly scaled simulated contact matrix $M_S' = \alpha M_S$ can be expressed as:

  $SSE(M_C, M_S') = SSE(M_C, \alpha M_S) = \sum_{i,j}\left(m_{ij}^C - \alpha m_{ij}^S\right)^2$ *. (Equation 1)*

  Although the unit of the SSE is number of contacts squared, we will treat it as a dimensionless number for brevity. Equation 1 is minimized at:

  $$\alpha = \frac{\sum_{i,j}\left(m_{ij}^C m_{ij}^S\right)}{\sum_{i,j}\left(m_{ij}^S\right)^2}.$$

  Calculating α yields the optimal average number of contacts in the ABM for settings such as households, without further substructure. However, for settings which have substructures, i.e., schools and workplaces, the simulated contact matrix is equal to the sum of the contact matrices of the substructures which can be scaled individually, i.e., each substructure has its own λ-value. Therefore, the SSE can be expressed as:

  $SSE(M_C, \sum_k \alpha_k M_S^k) = \sum_{i,j}\left(m_{ij}^C - \sum_k \alpha_k m_{ij}^{S,k}\right)^2$ *(Equation 2)*

  Here, M_S^k corresponds to the contact matrix of the k-th substructure, with their respective scaling factors α_k. In the following the α_k values for the substructures are obtained by numerically minimizing the Equation 2 using Nelder-Mead algorithm implemented by the Optim.jl package [37] in Julia [38]. To assess the numerical stability of the solutions we run the minimisation procedure 10,000 times with random initial conditions followed by a k-means clustering implemented in the Clustering.jl package in Julia [39]. This allows us to identify the different local minima and estimate the global minimum. For the further analysis, we use the global minimum to define the contact numbers for the respective substructures in the ABM.” [line 260-305].

- Contact sampling methodology: We clarified the rationale and procedure for sampling with replacement. Sampling with replacement is used for computational efficiency when simulating large populations while preserving the statistical properties of the empirical contact distribution. See:

  “The contact sampling for a specific individual I in the setting S_T of type T works in two steps. First, the number of contacts N_C the individual I will have is drawn from a Poisson distribution with mean λ_T, depending on the type T of the setting S_T. This means and distributions are based on the empirical data from COVIMOD so that the marginal number of contacts are

stable across approaches. Second, from the individuals present in the setting S_T excluding individual I, N_C individuals are randomly drawn with replacement and recorded as contacts. Sampling with replacement is performed to reduce computational complexity. For large settings such as municipalities, where only a small proportion of individuals present are selected as contacts, sampling without replacement would result in the computational cost scaling with the number of individuals present rather than the number of contacts sampled [28]. For our analysis, contacts are drawn for randomly selected individuals in the population until the total number of 10^7 recorded contacts are reached. This number of contacts was chosen as a cut-off to assure that uncertainties of the simulated contact behaviour are negligible for further analyses. [line 209-221].

- Contact matrix construction: We provided additional details on the construction of the contact matrix within our ABM. Specifically, we describe the construction process using the exactly known ages of both contact partners and grouping them into age-groups to derive the simulation contact matrices. See lines 216-220: *"For every selected individual all contacts they have during one day are sampled. Based on sampled contacts, we derive the simulated contact matrices. Since we know the exact ages of all contact partners, we can directly construct the contact matrices by grouping them into five-year age groups and calculating the average number of daily contacts between all age groups for every setting."* [line 221-226].

Together, these revisions provide a clearer, description of how contact parameters are calibrated, how contacts are sampled, and how matrices are constructed, thereby improving the transparency of our research.

Reviewer #3:

and how the hierarchical structure is built (no details are provided on the data sources used for building it, how it is implemented in practice, and how it affects contacts).

Response:

The hierarchical structure is a component of the synthetic population Gesyland. While our original manuscript included a short description on the synthetic population generation, we extended this description to include more details on the synthetic population construction. Specifically, we incorporated a description of the setting construction including the construction of the substructures for schools and workplaces. We also improved the references mentioned by adding a newly published article on the technical description of the construction of the synthetic population. The adapted part of the manuscript now read:

"The structure of contacts in GEMS is a result of the association of individuals to settings. The associations with settings are determined during the generation of the Gesyland population [29,30], a synthetic population that represents various characteristics of the German population. The locations and types of settings, such as home locations, schools or workplaces, are obtained from OpenStreetMap [31]. The home locations are filled with households, where the household size is obtained from the household size distribution based on the German Census 2011 [32]. This Census data is available at a 100m square grid, allowing for regional variations in household size distribution. Individuals in the households are created probabilistically based on demographic tables within household size classes. Further variables like education, occupation and health related variables obtained from the German microcensus 2019 [33] and the German health survey GEDA (2019/2020-EHIS) [34], and added via a predictive random forest procedure. The assignment of individuals to schools and workplaces is based on individual characteristics and the interregional commuting structure derived from commuting tables [35]. Substructures in

schools are created based on the structure of the school system. Schools consist of school years which consist of classes. Additionally multiple school may share the same common facilities and are structured in a school complex. For workplaces substructures are determined by randomly creating smaller groups of the workers in a workplace and thereby create departments and offices. Workplaces sharing the same location are grouped in a workplace site. While producing the individual properties and affiliations, the known constraints on higher levels are kept. These constraints included among others the total number of employed people in a region and the number of people having a certain educational level. Hence, the hierarchical population structure is consistent to known totals obtained from the German Census 2011 [32] regarding the households' structure, and consistent to estimated totals for all other variables according to the German microcensus 2019 [33] and German health survey GEDA (2019/2020-EHIS) [34]. A dedicated description of the Gesyland population and the method used for its creation elsewhere [30]." [line 227-252].

Reviewer #3:

The Appendix mentions social restrictions. However, the present study applies to a period without restrictions.

Response:

We thank the reviewer for this comment. The study period indeed corresponds to a time without formal social restrictions, and no restriction or NPI scenarios were simulated, calibrated, or analysed.

To avoid ambiguity, we have revised the appendix to make it clearer that the references to restrictions are only intended to motivate consideration of specific settings and do not form part of the present analysis. The changed text passages are as follows.

For Households:

"Compared to other types of contacts, household contacts are difficult to avoid because the shared living space can rarely be completely separated. Therefore, especially during periods of severe contact restriction, such as lockdowns, household contacts are critical to the pandemic's progression. This highlights the need for models to accurately reflect the household contact structures, especially for models that aim to investigate the effects of contact restrictions such as GEMS." [line 951-956].

For schools:

"Schools were subject to substantial restrictions in many countries. These restrictions were not enforced without reason as schools have been identified as significant contributors to the spread of pathogens [55]. Accurately representing the contact structure without restrictions is key to understanding how restrictions in schools can mitigate pathogen spread." [line 964-967].

And for other contacts:

"These types of contacts were the main targets of contact restrictions during the Covid-19 pandemic. However, it is almost impossible to completely ban these other contacts, as they include contacts that arise during essential activities, such as going to the supermarket or to the doctor. Therefore, an accurate parametrisation of other contacts is essential to model the disease spread with and without restrictions." [line 1009-1014].

Reviewer #3:

It is mentioned that the agent-based model is obtained from the Gesyland population. Even if this synthetic population was introduced somewhere else, essential information should be provided here to make the manuscript self-consistent.

Response:

While our original manuscript included a short description on the synthetic population generation, we extended this description to include more details on the synthetic population construction. Specifically, we incorporated information on the methods applied, the data sources used and the generated population. We also reference a newly published article including a detailed technical description of the methods employed for the construction of the synthetic Gesyland population. For the changes in the manuscript, we kindly refer the reviewer to the response to the hierarchical substructure comment above.

Reviewer #3:

The synthetic construction of realistic contact patterns and ways to improve within-setting mixing in agent-based models have been the subject of quite an amount of work already. It is not clear the contribution of the present study with respect to previous works. Some of these works are not cited (Fumanelli et al https://doi.org/10.1371/journal.pcbi.1002673, Mistry et al https://doi.org/10.1038/s41467-020-20544-y, Moreno Lopez et al DOI: 10.1126/sciadv.abd8750). Others are cited already (e.g. Hinch et al, Kerr et al, and Prem et al), but they are not properly discussed.

Response:

We thank the reviewer for these sources. We have now included the mentioned sources within our manuscript and added a paragraph within the introduction where we mention several of the sources you suggested to highlight similar approaches with different aims (mainly constructing contact networks or contact matrices based on synthetic populations):

"*While this question is particularly relevant to large-scale ABMs for infectious disease modelling, which usually rely on the assumption of random mixing to reduce computational complexity, it is also relevant in other fields [5,6]. Random mixing assumptions are commonly employed when constructing contact networks and matrices from synthetic population data [18–22]. Since such networks and matrices are used in infectious disease models, including compartmental and network models, assessing the validity of the random mixing assumption has implications for infectious disease modelling practice beyond large-scale ABMs.*" [line 103-109].

Further we mention these sources in the conclusion of our manuscript:

"*Although this study focuses on representing contact behaviour in ABMs, random mixing assumptions are also employed to construct contact matrices or networks [18–22]. These approaches generate synthetic populations and settings using population-specific datasets, such as census data, and define mixing behaviour within these settings. Based on these populations, age-stratified contact matrices or networks of the synthetic population are obtained. While some of these approaches also use a random mixing assumption, others incorporate more complex mixing behaviours, which could inform the development of ABMs. However, in ABMs contacts must be sampled at each time step, which requires efficient sampling methods. In contrast, contact matrices and networks are constructed once, corresponding to a single time step for the ABM. Nevertheless, these similar approaches may provide further insight into more*

refined contact sampling methods and help define metrics for comparing contact structures." [line 728-739].

Reviewer #3:

The analysis is poor and the results are not interpreted. For instance, authors mention that multiple waves of COVIMOD, where aggregated. It would be important to understand the differences from one wave to another. This would help gauge the discrepancy between modelled and empirical matrices. Also treating the same individuls in different waves as independent individuals could introduce a bias. This should be discussed.

Response:

We thank the reviewer for raising this point. Since all three waves were conducted within the same winter period (November 2022 to March 2023), we assumed that there were no significant variations in contact behaviour. Furthermore, as there were no restrictions during this period, changes in contact behaviour due to such restrictions are not applicable. We have clarified the aggregation and the reasoning behind the similarity in the description of the COVIMOD study.

"Since we want to consider the contact behaviour under non-restrictive conditions, we use the final three survey waves (wave 34 – 36), conducted between November 2022 and March 2023 and consider them as one large survey, treating participants who responded to multiple survey waves as independent participants. Since all survey waves were conducted during the same winter period and no contact restrictions were imposed, we neglected seasonal effects and other substantial changes in contact behaviour. These three waves capture the contacts of 7,481 participants, who reported a total of 23,146 individual contacts categorized as household (8,056), workplace (4,495), school (2,370) and other contacts (8,224), i.e., contacts the participant had in their household, workplace, school or in any other place, respectively." [line 129-137].

Although our assumption of independent participants introduces some bias, we believe it is still a sensible approach for our analysis. Of the participants, 2,006 contributed to a single survey, 1,134 contributed to two, and 1,069 contributed to all three of the analysed survey waves. Thus, approximately 3,200 participants were already included in the analysis due to prior participation in survey waves. Treating these individuals as independent participants introduces some bias. The repeated observations from the same individuals, for example, reduce the effective sample size, which could lead to an underestimation of the contact matrix uncertainty. However, given the constantly changing contact behaviour of individuals, the potentially different weekdays on which the waves were conducted, and our focus on population-level contact distributions rather than individual-level inference, we consider this bias to be acceptable for the purposes of our analysis.

To acknowledge these potential biases in the manuscript we extend the limitations of the COVIMOD dataset in the discussion:

"Another limitation arises from treating participants who contributed to multiple survey waves as independent observations. As approximately 2,200 participants took part in more than one survey, treating them as independent will introduce bias. This includes a reduction in the effective sample size and an underestimation of uncertainty due to more stable, repeated individual contact patterns assuming that participants retain some of their contact behaviour between survey waves. However, since our analysis focuses on population-level contact behaviour rather

than individual-level inference, we consider this bias to be acceptable for the purposes of this study." [line 659-666].

Reviewer #3:

Then, do the authors found any explanation on why the rescaling parameter, lambda, for schoolyear is the only one to be different from zero?

Response:

Thank you for raising this point. To improve the interpretability of the numerical minimisation procedure and to better understand why different λ-values emerge across sub-locations, we extended our approach by incorporating a k-means clustering of the solutions obtained from multiple optimisations using random initial conditions. Because the numerical minimisation can converge to local minima, clustering the resulting minima allowed us to systematically identify distinct groups of solutions, each representing a different local minimum with its own combination of rescaling parameters and associated SSE. This analysis revealed that several parameter combinations can fit the data reasonably well, but they differ in how they distribute λ across sub-locations. Importantly, the λ-hierarchy reported in the original manuscript corresponds to the global minimum: it is the solution most frequently recovered across runs and yields the lowest SSE. The alternative hierarchies arise from local minima but are less common and provide a worse fit. We report the clustering method in the methods section:

"To assess the numerical stability of the solutions we run the minimisation procedure 10,000 times with random initial conditions followed by a k-means clustering implemented in the Clustering.jl package in Julia [39]. This allows us to identify the different local minima and estimate the global minimum. For the further analysis, we use the global minimum to define the contact numbers for the respective substructures in the ABM." [line 287-291],

and the different possible combination of lambda-values and their respective frequency during the optimisation process in the results section for school contacts:

"The contact matrices for all substructures of the school using the uncalibrated random contact sampling are displayed in Figure 8 in the Appendix. Here, the school classes and school years represent highly age-assortative mixing while schools and school complexes also capture contacts between age groups. Calibration of the average number of contacts for the substructures yields: $\lambda_{schoolyear} = 1.26$ *and* $\lambda_{class} = \lambda_{school} = \lambda_{schoolcomplex} \approx 0$. *As expected, the age structure of school years alone most closely replicates the contact structure observed in COVIMOD. Note that these average numbers of contacts correspond to the most common solution of the numerical minimisation. Using the k-means cluster analysis of the minimisation results with random initial conditions we identified this kind of solution as the global minimum being obtained 88 % of times. The remaining minima correspond to local minima. Most common were combinations of school classes and school years, i.e., where* $\lambda_{class} \neq 0$ *and* $\lambda_{schoolyear} + \lambda_{class} \approx 1.26$. *Less than 0.3% of solutions also show contributions of the school setting. Further information on the identified clusters is displayed in Table 7 in the appendix. Looking at the values of Equation 2 for these solutions shows that* $\lambda_{schoolyear} = 1.26$ *is the global minimum, indicating that the small differences in the contact structures of the substructures leads to the best fit when only the schoolyear is included. In the following we use the contact numbers corresponding to this global minimum. The calibrated ABM school contact matrix is displayed in Figure 2 (B)."* [line 385-402],

and in the workplace section:

"The contact matrices for the four workplace substructures using the uncalibrated random contact sampling are presented in Figure 9 in the Appendix. As all substructures have a similar age structure, i.e., the age-constellation of workers in an office is on average identical to the age-constellation of entire companies, all substructures also exhibit a similar contact structure. The calibration of the contact numbers of the substructures with the COVIMOD data results in $\lambda_{workplace} = 0.29$, $\lambda_{department} = 0.81$ and $\lambda_{office} = \lambda_{workplacesite} \approx 0$. As for the school contacts, we used k-means clustering to identify that this solution corresponds to the global minimum of the SSE. It was obtained in 91% of the runs. Most remaining solutions correspond to other combinations of workplace and department with $\lambda_{workplace} + \lambda_{department} \approx 1.1$. In 3% of cases there are contributions from the office. Note, that the SSE differences between clusters displayed in Table 8 of the appendix are much smaller for the workplace substructures than for the school substructures due to the similarity of the age composition and contact structures across substructures. In the following we will again use this global minimum for our analysis." [line 428-440].

We present the raw clustering results as Tables 7 and 8 in the appendix.

Reviewer #3:

Finally, the epidemiological consequences of the discrepancies between real and modelled matrices are not analyzed, and, more in general the epidemiological significance of the work is not properly discussed. Authors should complement their analysis with epidemic simulations.

Response:

We thank the reviewer for this valuable suggestion. We agree that linking the identified matrix differences to epidemic outcomes is essential, and we have substantially revised the manuscript to address this point.

In the revised version, we added a new section "Epidemic Impact Analysis" (line 306-337) to the methods section, a section titled "Epidemic Impact Analysis Based on a Compartmental Model" (line 522 - 574) to the results section, and multiple paragraphs to the conclusion (line 601-621, line 698-711). Since these changes include entirely new sections and we want to keep this document at a readable length, we do not display the changes here but rather refer to the changed document lines. We quantify the epidemiological consequences of using the COVIMOD versus the ABM-derived (GEMS) contact matrices using the two methods suggested by the reviewer:

a. Next-generation matrix analysis: We compute and compare the basic reproduction numbers implied by each matrix in an age-structured SIR-model and report a disease-independent reproduction number ratio. This ratio is substantially above one, indicating that the two matrices lead to substantially different transmission dynamics.
b. Age-structured SIR simulations: In line with the reviewer's recommendation to use a compartmental model (and not the ABM) as the model, we implemented a simple age-structured SIR model in which the contact matrices determine age-group mixing. We examine key outcomes, including final attack rate, peak incidence, and age-specific dynamics. The matrices yield different epidemic outcomes and visibly different spread patterns through the age groups. Notably, after adjusting transmission so that both simulations have the same reproduction number, the ABM-based matrix produces a higher total number of infections.

Together, these analyses demonstrate that the observed structural differences between the survey- and ABM-based matrices can translate into substantial differences in disease dynamics within our utilised SIR-model. Our results also suggest that relying on random-mixing assumptions and calibrating solely to match empirical reproduction numbers could overestimate transmission potential. We believe these additions strengthen our manuscript, specifically the epidemiological interpretation of our findings.

Reviewer #3:

"It could also be helpful to use other types of contact data such as sensor data for calibration": This sentence is too vague. Authors should discuss what sensor data can be employed (e.g. RFID, maybe? see Cattuto et al https://doi.org/10.1371/journal.pone.0011596)

Response:

To address this comment, we clarified the sentence on sensor data. Specifically, we added more details about our suggested usage of sensor data and included the mentioned reference (among others). The changes include:

"Other types of contact data, such as sensor data, could be helpful for the calibration of contact behaviour. Sensor data, which is collected through devices that automatically record contacts based on proximity to other devices have been shown to record chance encounters more reliably compared to classical survey data, making it more suitable to parametrise random contacts [45,48]." [line 714-718].
