# Supplementary material for: A comparison of contact patterns derived from the population structure in agent-based models and empirical contact survey data
Source: PLoS Comput Biol. 2026 Jun 18;22(6):e1013533. doi: 10.1371/journal.pcbi.1013533 (PMC13309034; doi:10.1371/journal.pcbi.1013533)
Supplement: Attachment — Submitted filename: Revision Letter.docx [file pcbi.1013533.s003.docx]

### Point-by-point response

**Reviewer #1:**

I would like to thank the authors for carefully addressing my previous remarks and for extending the analysis to include epidemiological implications through the next-generation matrix approach and a compartmental model. This addition demonstrates the authors’ willingness to engage with the broader implications of their findings.

The new analyses clearly show that what appear to be relatively small structural differences between contact matrices reconstructed via the ABM approach and those derived from empirical data can translate into substantial epidemiological differences. However, I think that the interpretation provided by the authors warrants further reflection.

The manuscript suggests that although differences emerge in simpler modeling frameworks, these discrepancies may not necessarily persist within a full ABM setting. While this statement is conceptually valid, it appears to underestimate the structural role of contact matrices in shaping epidemic dynamics. If two contact matrices lead to divergent outcomes in a next-generation framework and in a compartmental model, it is unlikely that these differences would systematically disappear in a more complex ABM. On the contrary, structural discrepancies in mixing patterns are typically amplified rather than neutralized when embedded in higher-dimensional systems.

**Authors:**

We thank the reviewer for this comment. We have rephrased our discussion of the differences observed in the SIR model to reflect this suggestion. Specifically, we have reduced the emphasis on the limited transferability of these results to the full ABM. However, we maintain the important point that these results cannot be directly transferred and instead indicate potential differences. The revised paragraphs can be found in lines 602–620:

*The comparison of the resulting contact matrices using an age-structured SIR-model indicates that the differences in the contact matrices could translate into substantial differences in the epidemic outcomes. Using the next-generation matrix approach resulted in a reproduction number ratio substantially larger than one, corresponding to a faster disease spread when using the survey-based compared to the simulated contact matrix in the SIR-model. We also observed differences of the disease dynamics simulated in the SIR-model, including differences in outcomes such as the final attack rate and, peak infection numbers. In addition to these aggregate epidemiological indicators, the two contact matrices produced broadly similar but still noticeably different spreading dynamics between age groups. These differences became more apparent when both simulations were adjusted to equal reproduction numbers. We obtain a similar impact on the infectious disease simulation when we include group contacts.*

*Together these findings suggest that simplified algorithms for contact behaviour based only on population structure in ABMs may lead to unintended consequences when modelling infectious diseases. However, our comparison is based on a simple SIR model, which does not take into account factors such as contact clustering or repeated interactions which influence transmission dynamics both in the real-world and in ABMs. Therefore, while integrating both contact matrices into the SIR model yields different spreading dynamics and epidemic outcomes, estimating differences between real-world spreading dynamics and those in an ABM warrants further investigation.*

**Reviewer #1:**

This raises a central question that is not sufficiently addressed: how can these differences be reduced? The manuscript convincingly demonstrates that discrepancies exist, but it stops short of proposing or evaluating strategies to minimize them. There is already a substantial body of literature focused on aligning synthetically generated contact matrices with empirical data, including approaches such as those proposed by Willem1 and Fumanelli2 and colleagues. These contributions aim precisely at reducing the gap between model-generated and survey-based mixing patterns. A more explicit engagement with this literature would strengthen the discussion and provide readers with constructive pathways forward.

**Authors:**

We thank the reviewer for raising this important point. To address this issue, we have extended our discussion of potential methods to reduce the differences between the contact structures observed in COVIMOD and those generated in the ABM. In particular, we now discuss the inclusion of more granular settings as a potential approach, following the work by Willem et al. The second source provided by the reviewer is also highly relevant to our manuscript and is already cited in both the Introduction and the Discussion, as it follows a similar approach by constructing contact matrices based on public data for setting association and homogeneous mixing within settings.

The extended discussion of potential methods to reduce differences between real-world and simulated contact patterns can be found in lines 621–656:

*The discrepancies between the contact structures implied by random mixing within settings assumptions in an ABM and contact surveys could be reduced by employing more refined contact sampling methods. Potential approaches include a setting-size dependent number of contacts. For households, the correlation of increasing numbers of contacts with increasing household size has been demonstrated in [20]. However, it is not obvious to which extent the underestimation of child-child contacts is a result of the lack of household size dependence or actually a result of a higher number of contacts of children with their siblings. The latter has also been shown to be a determining factor for household contacts [43].*

*The inclusion of a setting-size-dependent number of contacts can be understood as the inclusion of more setting types with their own average number of contacts, i.e. households with a specific size (range) would correspond to a new setting type. The inclusion of further settings could be a potential solution to reduce differences between contact patterns in the real-world and in ABMs. Extending the implemented settings to include additional settings such as supermarkets or representing the existing settings on a more granular scale, for example, by replacing the large municipality settings with local communities [44]. This more granular scale could ensure that the arising contacts more closely represent the contact structure of the other contacts in COVIMOD. However, this approach requires additional data on the members of such settings which is rarely available. Otherwise setting association must be based on assumptions made by the modellers. While such approaches could introduce more granular settings they would continue to be based on homogeneous mixing within these settings.*

*The representation of heterogeneous contact behaviour requires methods that include household size dependent number of contacts and contact probabilities dependent on attributes of the potential contacts, such as their age. Similar attribute-based approaches could address the limitations in representing the other contacts. However, such attribute-based approaches would drastically increase the model’s computational complexity, as the probability of sampling an individual as a contact would depend on the properties of both contact partners. One simplified solution could be the implementation of two independent subsettings of which one captures age assortitiveness while the other does not.*

*Representing heterogeneous other contacts could also be achieved while maintaining the low computational complexity of random mixing within settings. Assuming a strong overlap between the other contacts and the contacts occurring in schools, workplaces or households a potential approach involves sampling additional contacts within these existing settings to represent the heterogeneous mixing observed in COVIMOD. Using this approach for the structure observed for the “other contacts” together with the currently used random municipality-based contacts could represent both random and structured other contacts.*

**Reviewer #1:**

In light of the new epidemiological analyses, the contribution of the manuscript also needs to be reconsidered in terms of its impact. The methodological framework is carefully structured and clearly explained. However, the finding that the reconstructed matrices lead to markedly different epidemic outcomes substantially limits the practical applicability of the current approach. Without demonstrating either (i) that these discrepancies can be mitigated, or (ii) that the ABM framework produces comparable epidemic results despite structural differences, the work remains primarily diagnostic rather than generative.

At present, the manuscript documents an important limitation of the modeling approach of the authors but does not yet provide sufficient guidance on how to overcome it or on when the approximation may still be appropriate. For this reason, and despite the authors’ substantial efforts to address previous comments, I find that in its current form the manuscript does not yet offer a sufficiently compelling advance for the broader computational epidemiology community.

1. Willem, L. et al. The impact of contact tracing and household bubbles on deconfinement strategies for COVID-19. Nat Commun 12, 1524 (2021).

2. Fumanelli, L., Ajelli, M., Manfredi, P., Vespignani, A. & Merler, S. Inferring the Structure of Social Contacts from Demographic Data in the Analysis of Infectious Diseases Spread. PLOS Computational Biology 8, e1002673 (2012).

**Authors:**

We agree that our analysis does not provide clear answer on how to accurately represent contact behaviour in large-scale ABMs. Our focus at this stage was on describing the differences and consequences of using contact sampling based on population structure widely used in ABMs. We highlight various potential approaches that could reduce the identified mismatches and should be assessed in additional studies, within the discussion section of the manuscript (see the previous comment).

Furthermore, we highlight that data collected in surveys have their own limitations. Therefore, simply aligning synthetic and empirical contact numbers from contact survey data might not be the ultimate goal. Further work in in the measurement of contact behaviour is needed, for example assessing the agreement in self-reported contacts and GPS-based estimations to clearly identify the contact structures that should be represented. In order to address this issue, we refer to the need of further research in the discussion section of the manuscript in lines 710-729:

As described above, the epidemic impact analysis performed has to be interpreted with caution. While we identify mismatches of the transmission dynamics when parametrising an SIR model with the COVIMOD-based vs the simulated contact matrix, these mismatches cannot be directly transferred to mismatches between real-world infections and infections simulated in the “full” ABM. Within our analysis we use the SIR model as an approximation of both the real-world and the ABM. This approach implicitly assumes that contact matrices alone suffice to represent infections and neglects features such as the clustering of contacts which we know to influence real-world and simulated disease dynamics. Furthermore, we assume that COVIMOD with its previously discussed limitations accurately captures all contacts relevant for disease transmission. We know that these assumptions are only approximations and do not hold exactly. Consequently, our results should be viewed as an indication that mismatches might exist. Further studies should accurately assess this indication by simulating different contact behaviours within the ABM to identify their potential impact on disease transmission.

Future research should refine contact sampling methods in ABMs to better represent the contact behaviour observed in the real-world, especially for studies focusing on disease dynamics in specific subgroups. Other types of contact data, such as sensor data, could be helpful for the calibration of contact behaviour. Sensor data, which is collected through devices that automatically record contacts based on proximity to other devices have been shown to record chance encounters more reliably compared to classical survey data, making it more suitable to parametrise random contacts [45,48].
