# Supplementary material for: A comparison of contact patterns derived from the population structure in agent-based models and empirical contact survey data
Source: PLoS Comput Biol. 2026 Jun 18;22(6):e1013533. doi: 10.1371/journal.pcbi.1013533 (PMC13309034; doi:10.1371/journal.pcbi.1013533)
Supplement: Peer review history

# Peer review: Decision Letter 0

**DOI:** 10.1371/journal.pcbi.1013533.r001

**Authors:** Nicola Perra, Denise Kühnert

31 Oct 2025

PCOMPBIOL-D-25-01881

A comparison of random mixing in a structured agentbased model with empirical contact survey data

PLOS Computational Biology

Dear Dr. Suer,

Thank you for submitting your manuscript to PLOS Computational Biology. After careful consideration, we feel that it has merit but does not fully meet PLOS Computational Biology's publication criteria as it currently stands. Therefore, we invite you to submit a revised version of the manuscript that addresses the points raised during the review process.

Please submit your revised manuscript within 60 days Dec 31 2025 11:59PM. If you will need more time than this to complete your revisions, please reply to this message or contact the journal office at ploscompbiol@plos.org. When you're ready to submit your revision, log on to https://www.editorialmanager.com/pcompbiol/ and select the 'Submissions Needing Revision' folder to locate your manuscript file.

Please include the following items when submitting your revised manuscript:

* A rebuttal letter that responds to each point raised by the editor and reviewer(s). You should upload this letter as a separate file labeled 'Response to Reviewers'. This file does not need to include responses to formatting updates and technical items listed in the 'Journal Requirements' section below.

* A marked-up copy of your manuscript that highlights changes made to the original version. You should upload this as a separate file labeled 'Revised Manuscript with Track Changes'.

* An unmarked version of your revised paper without tracked changes. You should upload this as a separate file labeled 'Manuscript'.

If you would like to make changes to your financial disclosure, competing interests statement, or data availability statement, please make these updates within the submission form at the time of resubmission. Guidelines for resubmitting your figure files are available below the reviewer comments at the end of this letter

We look forward to receiving your revised manuscript.

Kind regards,

Nicola Perra

Academic Editor

PLOS Computational Biology

Denise Kühnert

Section Editor

PLOS Computational Biology

**Journal Requirements:**

1) We ask that a manuscript source file is provided at Revision. Please upload your manuscript file as a .doc, .docx, .rtf or .tex. If you are providing a .tex file, please upload it under the item type u2018LaTeX Source Fileu2019 and leave your .pdf version as the item type u2018Manuscriptu2019.

2) Please upload all main figures as separate Figure files in .tif or .eps format. For more information about how to convert and format your figure files please see our guidelines:

https://journals.plos.org/ploscompbiol/s/figures

**Reviewers' comments:**

Reviewer's Responses to Questions

**Comments to the Authors:**

**Please note here if the review is uploaded as an attachment.**

Reviewer #1: This manuscript presents a thoughtful and well-executed comparison between contact structures simulated by an agent-based model (GEMS) and those observed in the empirical COVIMOD contact survey. By evaluating how the random mixing assumption performs across different settings—households, schools, workplaces, and other environments—the study contributes to improving the realism and interpretability of epidemic models. The work addresses a timely and relevant topic for computational epidemiology. However, several methodological and interpretative aspects could be clarified or extended to strengthen the conclusions. Addressing these points would make the manuscript a more robust and impactful contribution to the literature.

I list my comments here below, listed in order of importance and divided between “major” and “minor”.

Major Comments

1) The authors rightfully acknowledge that their method does not incorporate age-specific differences in contact activity and that this is a big limitations of their work. Since such heterogeneity is a fundamental feature of human contact patterns, its omission substantially limits the model’s realism. The authors could explore introducing an age-dependent calibration factor for contact frequency. This extension (although more parameterized) should be computationally manageable and could greatly improve model fidelity. If implementing this is not feasible, it would be helpful to explain the technical constraints preventing it.

2) The decision to compare empirical and simulated contact matrices is appropriate and informative. However, the interpretation remains largely structural, without exploring how these differences may affect epidemic potential. To strengthen the manuscript’s epidemiological relevance, I encourage the authors to use a next-generation matrix approach to derive and compare the basic reproduction numbers (R₀) from the COVIMOD and GEMS matrices. This addition would contextualize the observed discrepancies in a more interpretable and policy-relevant way, since identical matrix differences can have markedly different effects on R₀ depending on the age pairs involved. It could be that the discrepancies observed between COVIMOD and GERMS matrices do not have a striking epidemic impact.

3) The imputation of missing ages for group contacts using the national population age distribution is a practical choice, but location-specific age structures might provide a more accurate representation. I suggest reporting the proportion of group versus individual contacts (to put this problem in perspective) and exploring how alternative imputation strategies (for instance, setting-specific age structures) influence the results.

4) The assumption that all sub-locations (e.g., school classes, years) are equally likely contact environments simplifies reality and may overlook important structural heterogeneity. For example, contact intensity is typically highest within classes and lower between them (so the lambda for classes should be larger than the one for the year, contrary to the current results). Furthermore, the calibration procedure leads to hierarchical λ values across sub-locations, although their age structures appear similar. It would be helpful for the authors to elaborate on why this hierarchy arises, since I would expect all sub-locations lambdas to be equal, if the underlying structure of mixing at random is the same.

Minor Comments

1) Line 190: The period should be replaced by a comma; otherwise, the clause beginning with “using” lacks a clear subject.

2) The manuscript reports small deviations for home, school, and workplace settings. Could this partly reflect the smaller number of contacts in these settings compared to “other” contacts? Reporting relative errors, in addition to absolute differences, could clarify this point and might provide a better sense of the epidemiological significance of these discrepancies (see major comment 2).

In conclusion, this is an important and promising contribution to the ongoing discussion on how best to represent contact structures in large-scale agent-based models. By expanding the paper to quantify epidemiological consequences, incorporating age-specific contact activity, and clarifying several modelling assumptions, the paper would achieve a stronger impact and broader relevance for the field.

Reviewer #2: Thanks for the opportunity to review this manuscript.

In their work, the authors compare the contact matrices obtained from a large contact study in Germany between 2022 and 2023, with the contact matrices obtained from a synthetic population of Germany, used in a publicly available agent-based model.

The work is interesting, and it addresses an important research question. There is much research work that compares agent-based models and survey contact matrices, however, most of these studies date back to the POLYMOD study (used as a benchmark). Few studies have tackled this problem after COVID-19 and I see this paper as a nice contribution.

I have one major remark that I consider important to address and a minor comment. In general, my main concern is that the paper does not engage sufficiently with the consequences of its findings on the epidemiology of respiratory infections, and disease dynamics.

Major issues:

- The study exposes in detail the differences between contact matrices from survey and ABM but a fundamental question remains unanswered: what is the impact of such (relatively small differences) on simulated epidemics? This is a key question that naturally arises from the results. More specifically, I suggest comparing the results of a simulated SEIR stochastic model that uses either matrix, for different assumptions on disease transmissibility. Does the final attack rate varies significantly? Please, note that I am not suggesting using the ABM as the epidemic tool. Instead, simulate an age-structured compartmental model with different matrices.

- Related to the above point, the contact matrix can be directly linked to the basic reproductive number, R_0, in a model through its spectral radius. Are the spectral radiuses of the matrices very different? What is the difference in R_0 that we expect to observe given the different matrices?

Minor issues:

- On what data the synthetic population of GEMS is based? Is census data from the latest national census? What year does it represent? I might have missed this information from the paper. Results could be partially explained by the recency of the ABM population data.

Reviewer #3: The manuscript by Suer et al addresses on what extent the contact behavior modeled by agent-based model well reproduce real data. Agent based models build a synthetic population which capture the collocation of individuals in same settings – households, workplaces, schools, and other settings. The manuscript addresses the comparison between modelled and empirical contacts, where the latters are obtained from national surveys. It shows that modelled contacts in setting characterized by a well-defined age composition reproduce the basic properties of real patterns. Contacts outside households, schools, and workplaces, present the largest deviation with empirical data.

The manuscript is not clearly written. The importance of the work relative to the existing literature is not discussed. Finally, the analysis is poor. Therefore, I cannot recommend the publication of the work in the present form. Substantial revisions are necessary before the work can be considered suitable for publication.

More specifically:

The manuscript is not clearly written. Sentences are unclear in many parts. The analysis done is not clearly presented. Not enough background is provided on the agent-based model used in the study; therefore, it is not clear how the matrices are reconstructed and calibrated from the data. For instance, I could not understand the procedure for contact sampling in GEMS described in line 170-179 (why contacts are drawn with replacement?), and how the hierarchical structure is built (no details are provided on the data sources used for building it, how it is implemented in practice, and how it affects contacts). The Appendix mentions social restrictions. However, the present study applies to a period without restrictions. It is mentioned that the agent-based model is obtained from the Gesyland population. Even if this synthetic population was introduced somewhere else, essential information should be provided here to make the manuscript self-consistent.

The synthetic construction of realistic contact patterns and ways to improve within-setting mixing in agent-based models have been the subject of quite an amount of work already. It is not clear the contribution of the present study with respect to previous works. Some of these works are not cited (Fumanelli et al https://doi.org/10.1371/journal.pcbi.1002673, Mistry et al https://doi.org/10.1038/s41467-020-20544-y, Moreno Lopez et al DOI: 10.1126/sciadv.abd8750). Others are cited already (e.g. Hinch et al, Kerr et al, and Prem et al), but they are not properly discussed.

The analysis is poor and the results are not interpreted. For instance, authors mention that multiple waves of COVIMOD, where aggregated. It would be important to understand the differences from one wave to another. This would help gauge the discrepancy between modelled and empirical matrices. Also treating the same individuls in different waves as independent individuals could introduce a bias. This should be discussed. Then, do the authors found any explanation on why the rescaling parameter, lambda, for schoolyear is the only one to be different from zero? Finally, the epidemiological consequences of the discrepancies between real and modelled matrices are not analyzed, and, more in general the epidemiological significance of the work is not properly discussed. Authors should complement their analysis with epidemic simulations.

“It could also be helpful to use other types of contact data such as sensor data for calibration”: This sentence is too vague. Authors should discuss what sensor data can be employed (e.g. RFID, maybe? see Cattuto et al https://doi.org/10.1371/journal.pone.0011596)

**********

**Have the authors made all data and (if applicable) computational code underlying the findings in their manuscript fully available?**

The PLOS Data policy requires authors to make all data and code underlying the findings described in their manuscript fully available without restriction, with rare exception (please refer to the Data Availability Statement in the manuscript PDF file). The data and code should be provided as part of the manuscript or its supporting information, or deposited to a public repository. For example, in addition to summary statistics, the data points behind means, medians and variance measures should be available. If there are restrictions on publicly sharing data or code —e.g. participant privacy or use of data from a third party—those must be specified.

Reviewer #1: Yes

Reviewer #2: Yes

Reviewer #3: Yes

**********

PLOS authors have the option to publish the peer review history of their article (what does this mean?). If published, this will include your full peer review and any attached files.

If you choose “no”, your identity will remain anonymous but your review may still be made public.

**Do you want your identity to be public for this peer review?**   For information about this choice, including consent withdrawal, please see our Privacy Policy.

Reviewer #1: No

Reviewer #2: No

Reviewer #3: No

[NOTE: If reviewer comments were submitted as an attachment file, they will be attached to this email and accessible via the submission site. Please log into your account, locate the manuscript record, and check for the action link "View Attachments". If this link does not appear, there are no attachment files.]

**Figure resubmission:**

While revising your submission, we strongly recommend that you use PLOS’s NAAS tool (https://ngplosjournals.pagemajik.ai/artanalysis) to test your figure files. NAAS can convert your figure files to the TIFF file type and meet basic requirements (such as print size, resolution), or provide you with a report on issues that do not meet our requirements and that NAAS cannot fix.

After uploading your figures to PLOS’s NAAS tool - https://ngplosjournals.pagemajik.ai/artanalysis, NAAS will process the files provided and display the results in the "Uploaded Files" section of the page as the processing is complete. If the uploaded figures meet our requirements (or NAAS is able to fix the files to meet our requirements), the figure will be marked as "fixed" above. If NAAS is unable to fix the files, a red "failed" label will appear above. When NAAS has confirmed that the figure files meet our requirements, please download the file via the download option, and include these NAAS processed figure files when submitting your revised manuscript.

**Reproducibility:**

To enhance the reproducibility of your results, we recommend that authors of applicable studies deposit laboratory protocols in protocols.io, where a protocol can be assigned its own identifier (DOI) such that it can be cited independently in the future. Additionally, PLOS ONE offers an option to publish peer-reviewed clinical study protocols. Read more information on sharing protocols at https://plos.org/protocols?utm_medium=editorial-email&utm_source=authorletters&utm_campaign=protocols

# Author comment: Author response to Decision Letter 1

**DOI:** 10.1371/journal.pcbi.1013533.r002

17 Feb 2026

Attachment Submitted filename: revision_letter.pdf

# Peer review: Decision Letter 1

**DOI:** 10.1371/journal.pcbi.1013533.r003

**Authors:** Nicola Perra, Denise Kühnert

23 Mar 2026

PCOMPBIOL-D-25-01881R1

A comparison of contact patterns derived from the population structure in agent-based models and empirical contact survey data

PLOS Computational Biology

Dear Dr. Suer,

Thank you for submitting your manuscript to PLOS Computational Biology. After careful consideration, we feel that it has merit but does not fully meet PLOS Computational Biology's publication criteria as it currently stands. Therefore, we invite you to submit a revised version of the manuscript that addresses the points raised during the review process.

Please submit your revised manuscript by May 23 2026 11:59PM. If you will need more time than this to complete your revisions, please reply to this message or contact the journal office at ploscompbiol@plos.org. When you're ready to submit your revision, log on to https://www.editorialmanager.com/pcompbiol/ and select the 'Submissions Needing Revision' folder to locate your manuscript file.

Please include the following items when submitting your revised manuscript:

* A letter that responds to each point raised by the editor and reviewer(s). You should upload this letter as a separate file labeled 'Response to Reviewers'. This file does not need to include responses to formatting updates and technical items listed in the 'Journal Requirements' section below.

* A marked-up copy of your manuscript that highlights changes made to the original version. You should upload this as a separate file labeled 'Revised Manuscript with Track Changes'.

* An unmarked version of your revised paper without tracked changes. You should upload this as a separate file labeled 'Manuscript'.

If you would like to make changes to your financial disclosure, competing interests statement, or data availability statement, please make these updates within the submission form at the time of resubmission. Guidelines for resubmitting your figure files are available below the reviewer comments at the end of this letter

We look forward to receiving your revised manuscript.

Kind regards,

Nicola Perra

Academic Editor

PLOS Computational Biology

Denise Kühnert

Section Editor

PLOS Computational Biology

**Additional Editor Comments:**

Many thanks for the great work done in the first round of revisions. As you can see the first reviewer raises some interesting points which I think should be at least commented/explored in the discussion.

**Journal Requirements:**

If the reviewer comments include a recommendation to cite specific previously published works, please review and evaluate these publications to determine whether they are relevant and should be cited. There is no requirement to cite these works unless the editor has indicated otherwise.

1) We note that your Contact Structure Comparison Clean.pdf, and Contact Structure Comparison Clean.docx files are duplicated on your submission. Please remove any unnecessary or old files from your revision, and make sure that only those relevant to the current version of the manuscript are included.

2) Please provide an Author Summary. This should appear in your manuscript between the Abstract (if applicable) and the Introduction, and should be 150-200 words long. The aim should be to make your findings accessible to a wide audience that includes both scientists and non-scientists. Sample summaries can be found on our website under Submission Guidelines:

https://journals.plos.org/ploscompbiol/s/submission-guidelines#loc-parts-of-a-submission

**Reviewers' comments:**

Reviewer's Responses to Questions

**Comments to the Authors:**

**Please note here if the review is uploaded as an attachment.**

Reviewer #1: I would like to thank the authors for carefully addressing my previous remarks and for extending the analysis to include epidemiological implications through the next-generation matrix approach and a compartmental model. This addition demonstrates the authors’ willingness to engage with the broader implications of their findings.

The new analyses clearly show that what appear to be relatively small structural differences between contact matrices reconstructed via the ABM approach and those derived from empirical data can translate into substantial epidemiological differences. However, I think that the interpretation provided by the authors warrants further reflection.

The manuscript suggests that although differences emerge in simpler modeling frameworks, these discrepancies may not necessarily persist within a full ABM setting. While this statement is conceptually valid, it appears to underestimate the structural role of contact matrices in shaping epidemic dynamics. If two contact matrices lead to divergent outcomes in a next-generation framework and in a compartmental model, it is unlikely that these differences would systematically disappear in a more complex ABM. On the contrary, structural discrepancies in mixing patterns are typically amplified rather than neutralized when embedded in higher-dimensional systems.

This raises a central question that is not sufficiently addressed: how can these differences be reduced? The manuscript convincingly demonstrates that discrepancies exist, but it stops short of proposing or evaluating strategies to minimize them. There is already a substantial body of literature focused on aligning synthetically generated contact matrices with empirical data, including approaches such as those proposed by Willem1 and Fumanelli2 and colleagues. These contributions aim precisely at reducing the gap between model-generated and survey-based mixing patterns. A more explicit engagement with this literature would strengthen the discussion and provide readers with constructive pathways forward.

In light of the new epidemiological analyses, the contribution of the manuscript also needs to be reconsidered in terms of its impact. The methodological framework is carefully structured and clearly explained. However, the finding that the reconstructed matrices lead to markedly different epidemic outcomes substantially limits the practical applicability of the current approach. Without demonstrating either (i) that these discrepancies can be mitigated, or (ii) that the ABM framework produces comparable epidemic results despite structural differences, the work remains primarily diagnostic rather than generative.

At present, the manuscript documents an important limitation of the modeling approach of the authors but does not yet provide sufficient guidance on how to overcome it or on when the approximation may still be appropriate. For this reason, and despite the authors’ substantial efforts to address previous comments, I find that in its current form the manuscript does not yet offer a sufficiently compelling advance for the broader computational epidemiology community.

1. Willem, L. et al. The impact of contact tracing and household bubbles on deconfinement strategies for COVID-19. Nat Commun 12, 1524 (2021).

2. Fumanelli, L., Ajelli, M., Manfredi, P., Vespignani, A. & Merler, S. Inferring the Structure of Social Contacts from Demographic Data in the Analysis of Infectious Diseases Spread. PLOS Computational Biology 8, e1002673 (2012).

Reviewer #2: I thank the authors for addressing my remarks in their revision. All my comments have been addressed.

Reviewer #3: Authors have substantially revised the manuscript and addressed my comments in a satisfactory way. I believe the manuscript has improved and I recommend its acceptance.

**********

**Have the authors made all data and (if applicable) computational code underlying the findings in their manuscript fully available?**

The PLOS Data policy requires authors to make all data and code underlying the findings described in their manuscript fully available without restriction, with rare exception (please refer to the Data Availability Statement in the manuscript PDF file). The data and code should be provided as part of the manuscript or its supporting information, or deposited to a public repository. For example, in addition to summary statistics, the data points behind means, medians and variance measures should be available. If there are restrictions on publicly sharing data or code —e.g. participant privacy or use of data from a third party—those must be specified.

Reviewer #1: Yes

Reviewer #2: Yes

Reviewer #3: Yes

**********

PLOS authors have the option to publish the peer review history of their article (what does this mean?). If published, this will include your full peer review and any attached files.

If you choose “no”, your identity will remain anonymous but your review may still be made public.

**Do you want your identity to be public for this peer review?**   For information about this choice, including consent withdrawal, please see our Privacy Policy.

Reviewer #1: No

Reviewer #2: No

Reviewer #3: No

**Figure resubmission:**

While revising your submission, we strongly recommend that you use PLOS’s NAAS tool (https://ngplosjournals.pagemajik.ai/artanalysis) to test your figure files. NAAS can convert your figure files to the TIFF file type and meet basic requirements (such as print size, resolution), or provide you with a report on issues that do not meet our requirements and that NAAS cannot fix.

After uploading your figures to PLOS’s NAAS tool - https://ngplosjournals.pagemajik.ai/artanalysis, NAAS will process the files provided and display the results in the 'Uploaded Files' section of the page as the processing is complete. If the uploaded figures meet our requirements (or NAAS is able to fix the files to meet our requirements), the figure will be marked as 'fixed' above. If NAAS is unable to fix the files, a red 'failed' label will appear above. When NAAS has confirmed that the figure files meet our requirements, please download the file via the download option, and include these NAAS processed figure files when submitting your revised manuscript.

**Reproducibility:**

To enhance the reproducibility of your results, we recommend that authors of applicable studies deposit laboratory protocols in protocols.io, where a protocol can be assigned its own identifier (DOI) such that it can be cited independently in the future. Additionally, PLOS ONE offers an option to publish peer-reviewed clinical study protocols. Read more information on sharing protocols at https://plos.org/protocols?utm_medium=editorial-email&utm_source=authorletters&utm_campaign=protocols

# Author comment: Author response to Decision Letter 2

**DOI:** 10.1371/journal.pcbi.1013533.r004

22 May 2026

Attachment Submitted filename: Revision Letter.docx

# Peer review: Decision Letter 2

**DOI:** 10.1371/journal.pcbi.1013533.r005

**Authors:** Nicola Perra, Denise Kühnert

8 Jun 2026

Dear Mr. Suer,

We are pleased to inform you that your manuscript 'A comparison of contact patterns derived from the population structure in agent-based models and empirical contact survey data' has been provisionally accepted for publication in PLOS Computational Biology.

Before your manuscript can be formally accepted you will need to complete some formatting changes, which you will receive in a follow up email. A member of our team will be in touch with a set of requests.

Please note that your manuscript will not be scheduled for publication until you have made the required changes, so a swift response is appreciated.

IMPORTANT: The editorial review process is now complete. PLOS will only permit corrections to spelling, formatting or significant scientific errors from this point onwards. Requests for major changes, or any which affect the scientific understanding of your work, will cause delays to the publication date of your manuscript.

Should you, your institution's press office or the journal office choose to press release your paper, you will automatically be opted out of early publication. We ask that you notify us now if you or your institution is planning to press release the article. All press must be co-ordinated with PLOS.

Thank you again for supporting Open Access publishing; we are looking forward to publishing your work in PLOS Computational Biology.

Best regards,

Nicola Perra

Academic Editor

PLOS Computational Biology

Denise Kühnert

Section Editor

PLOS Computational Biology

***********************************************************

Reviewer's Responses to Questions

**Comments to the Authors:**

**Please note here if the review is uploaded as an attachment.**

Reviewer #1: I thank the authors for carefully taking into consideration my comments.

**********

**Have the authors made all data and (if applicable) computational code underlying the findings in their manuscript fully available?**

The PLOS Data policy requires authors to make all data and code underlying the findings described in their manuscript fully available without restriction, with rare exception (please refer to the Data Availability Statement in the manuscript PDF file). The data and code should be provided as part of the manuscript or its supporting information, or deposited to a public repository. For example, in addition to summary statistics, the data points behind means, medians and variance measures should be available. If there are restrictions on publicly sharing data or code —e.g. participant privacy or use of data from a third party—those must be specified.

Reviewer #1: Yes

**********

PLOS authors have the option to publish the peer review history of their article (what does this mean?). If published, this will include your full peer review and any attached files.

If you choose “no”, your identity will remain anonymous but your review may still be made public.

**Do you want your identity to be public for this peer review?**   For information about this choice, including consent withdrawal, please see our Privacy Policy.

Reviewer #1: No

# Editor report: Acceptance letter

**DOI:** 10.1371/journal.pcbi.1013533.r006

**Authors:** Nicola Perra, Denise Kühnert

PCOMPBIOL-D-25-01881R2

A comparison of contact patterns derived from the population structure in agent-based models and empirical contact survey data

Dear Dr Suer,

I am pleased to inform you that your manuscript has been formally accepted for publication in PLOS Computational Biology. Your manuscript is now with our production department and you will be notified of the publication date in due course.

The corresponding author will soon be receiving a typeset proof for review, to ensure errors have not been introduced during production. Please review the PDF proof of your manuscript carefully, as this is the last chance to correct any errors. Please note that major changes, or those which affect the scientific understanding of the work, will likely cause delays to the publication date of your manuscript.

Soon after your final files are uploaded, unless you have opted out, the early version of your manuscript will be published online. The date of the early version will be your article's publication date. The final article will be published to the same URL, and all versions of the paper will be accessible to readers.

For Research, Software, and Methods articles, you will receive an invoice from PLOS for your publication fee after your manuscript has reached the completed accept phase. If you receive an email requesting payment before acceptance or for any other service, this may be a phishing scheme. Learn how to identify phishing emails and protect your accounts at https://explore.plos.org/phishing.

Thank you again for supporting PLOS Computational Biology and open-access publishing. We are looking forward to publishing your work!

With kind regards,

Zsofia Freund

PLOS Computational Biology | Carlyle House, Carlyle Road, Cambridge CB4 3DN | United Kingdom ploscompbiol@plos.org | Phone +44 (0) 1223-442824 | ploscompbiol.org | @PLOSCompBiol
